# Genome Mining, Microbial Interactions, and Molecular Networking Reveals New Dibromoalterochromides from Strains of *Pseudoalteromonas* of Coiba National Park-Panama

**DOI:** 10.3390/md18090456

**Published:** 2020-09-03

**Authors:** Librada A. Atencio, Cristopher A. Boya P., Christian Martin H., Luis C. Mejía, Pieter C. Dorrestein, Marcelino Gutiérrez

**Affiliations:** 1 Centro de Biodiversidad y Descubrimiento de Drogas, Instituto de Investigaciones Científicas y Servicios de Alta Tecnología (INDICASAT), Clayton, Panama City 0843-01103, Panama; latencio@indicasat.org.pa (L.A.A.); CBoya@indicasat.org.pa (C.A.B.P.); christian.martin.hdz@gmail.com (C.M.H.); 2 Department of Biotechnology, Acharya Nagarjuna University, Nagarjuna Nagar, Guntur 522510, India; 3 Smithsonian Tropical Research Institute, Balboa Ancón, Panama City 0843-03092, Panama; 4 Collaborative Mass Spectrometry Innovation Center, Skaggs School of Pharmacy and Pharmaceutical Sciences, University of California San Diego, La Jolla, CA 92093, USA; pdorrestein@health.ucsd.edu

**Keywords:** *Pseudoalteromonas*, bromoalterochromides, antimicrobials, genome mining, biosynthetic gene cluster, MS/MS molecular networking, Coiba National Park

## Abstract

The marine bacterial genus *Pseudoalteromonas* is known for their ability to produce antimicrobial compounds. The metabolite-producing capacity of *Pseudoalteromonas* has been associated with strain pigmentation; however, the genomic basis of their antimicrobial capacity remains to be explained. In this study, we sequenced the whole genome of six *Pseudoalteromonas* strains (three pigmented and three non-pigmented), with the purpose of identifying biosynthetic gene clusters (BGCs) associated to compounds we detected via microbial interactions along through MS-based molecular networking. The genomes were assembled and annotated using the SPAdes and RAST pipelines and mined for the identification of gene clusters involved in secondary metabolism using the antiSMASH database. Nineteen BGCs were detected for each non-pigmented strain, while more than thirty BGCs were found for two of the pigmented strains. Among these, the groups of genes of nonribosomal peptide synthetases (NRPS) that code for bromoalterochromides stand out the most. Our results show that all strains possess BGCs for the production of secondary metabolites, and a considerable number of distinct polyketide synthases (PKS) and NRPS clusters are present in pigmented strains. Furthermore, the molecular networking analyses revealed two new molecules produced during microbial interactions: the dibromoalterochromides D/D’ (**11–12**).

## 1. Introduction

Marine natural products-based drug discovery was initially focused on macroorganisms such as algae, corals, sponges, and mollusks [1]. However, this approach has gradually expanded to the study of microorganisms associated to invertebrates and other animals that constitute a large portion of marine biodiversity. In particular octocoral-associated microbes, a prolific source of natural products with fascinating and unusual chemical structures and bioactivities, have become of interest to many drug discovery programs [2,3,4]. Currently, octocoral-associated bacteria reported for producing bioactive compounds belong to the genera *Streptomyces* (Actinobacteria), *Bacillus* (Firmicutes), *Vibrio*, and *Pseudoalteromonas* (Gammaproteobacteria), all obtained in culture.

Species of *Pseudoalteromonas* have an average genome size around 4.8 Mb [5], and studies regarding their genetic composition, ecology, and its relevance as a source of bioactive compounds have been increasing recently [6,7,8,9]. Species of this genus have been widely reported for the production of antimicrobial metabolites [6,7]. In particular species of *Pseudoalteromonas* that produce antimicrobials have been found in association with marine invertebrates [7]. Some studies have suggested that these bacteria play an important ecological role by providing their host with protection against pathogens through the formation of biofilms [6] and the production of antimicrobial metabolites [6,7,8,9]. Additionally, it is thought that species within the genus *Pseudoalteromonas* play an essential role in homeostasis of holobionts by means of their metabolic activities [7]. In a study carried out by Moree and collaborators, microbial interaction studies using MALDI IMS revealed how the strain *Pseudoalteromonas* sp. OT-59 displayed light-dependent antifungal properties against *Penicillium citrinum* isolated from an octocoral [9]. In a similar experiment *Pseudoalteromonas* sp. OT-59 also showed antibacterial activity against a strain of *Bacillus* [10].

In *Pseudoalteromonas*, antibacterial and antifungal properties have been correlated to their metabolite-producing capacity in pigmented strains. Though observed less frequently, non-pigmented strains are also potential metabolite producers [7,11,12]. The natural products biosynthesized by these microorganisms comprise alkaloids, polyketides, terpenoids, bacteriocins and siderophores with a wide range of bioactivities including antimicrobial, antiprotozoal, and cytotoxic [2,6,7].

Natural products biosynthesis research is undertaking a broad transformation, driven by technological developments in genomics, bioinformatics and analytical chemistry. Currently, it has become possible to computationally identify thousands of biosynthetic gene clusters (BGCs) in genome sequences, and to characterize them systematically. These BGCs are defined as physically clustered group of two or more genes in a particular genome that together encode for a biosynthetic pathway for the production of specialized metabolites [13,14,15]. Most of the natural products identified are produced by nonribosomal peptide synthetases (NRPSs) and/or polyketide synthases (PKSs).

The development of new genomic tools is revealing an untapped richness of secondary metabolites, larger than reported or expected for microorganisms in particular [4,16,17,18]. Studying these secondary metabolites, the Global Natural Products Social molecular networking web-platform (GNPS) is emerging as a powerful tool for the identification and dereplication of entire families of natural products [19]. GNPS allows the exploration of the chemical space of biological samples by allowing comparisons among MS^2^ spectra. Such comparisons consider the fragmentation patterns of molecules for clustering related-molecules in a spectral network. Thus, it is possible to study the nature and dynamics of the production of natural products in single microbes and during microbial interactions [19,20,21,22].

This work is focused in the study of biosynthetic gene clusters involved in the production of secondary metabolites of six *Pseudoalteromonas* strains—pigmented and non-pigmented—isolated from different species of octocorals from Coiba National Park, a marine protected area at the Pacific of Panama, designated by UNESCO as World Heritage Site since 2005.

**Figure 1:**
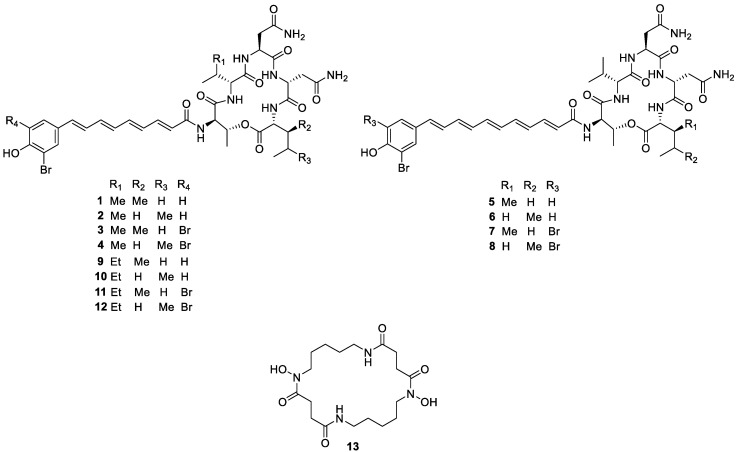
Structure of compounds **1**–**13** annotated by the Global Natural Products Social molecular networking web-platform (GNPS).

Herein we studied the genomes of six strains of *Pseudoalteromonas* and found high genetic potential for the production of secondary metabolites in both, non-pigmented and pigmented strains. We cultured *Pseudoalteromonas* strains from different species of octocorals (Table 1) collected in the waters of Coiba National Park. By performing molecular networking analysis, we detected and identified a series of metabolites (Figure 1) produced by *Pseudoalteromonas* when growing in monoculture as well as in co-culture with strains of *Staphylococcus aureus*, *Escherichia coli*, *Candida albicans*, and *Aspergillus fumigatus*.

## 2. Results

### 2.1. Genome Assembly and Annotation

**Figure 2:**
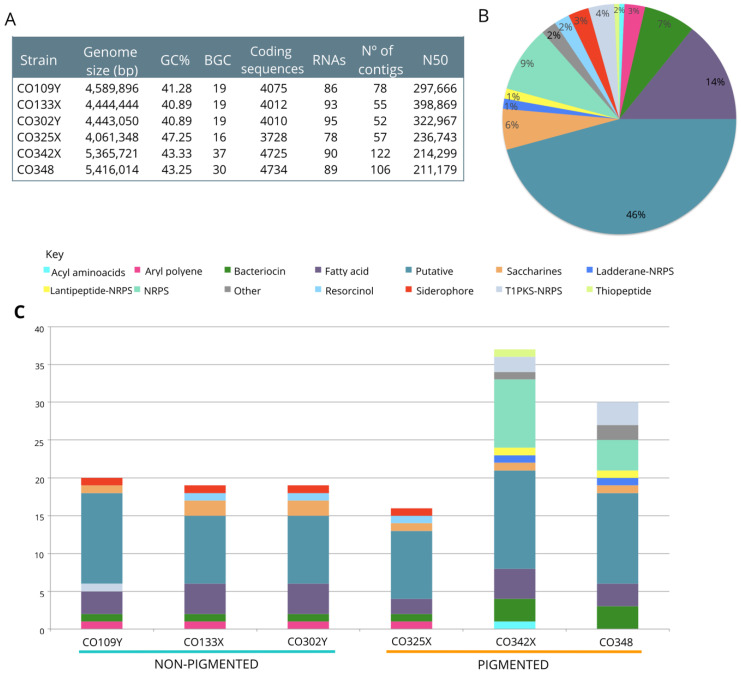
(**A**) *Pseudoalteromonas* genome statistics and summary of identified biosynthetic gene clusters (BGCs) across all genomes. (**B**) Overall distribution of BGC categories in all strains identified by antiSMASH. (**C**) Composition of BGC categories per genome, showing considerable variation between strains.

The whole genome of six *Pseudoalteromonas* strains were sequenced and functionally annotated and their genome size varied between 4.0 and 5.4 Mb. The genomes of non-pigmented strains (CO109Y, CO133X, CO302Y) varied between 4.4 and 4.5 Mb, while the genomes of pigmented (CO325X, CO342X and CO348) strains ranged from 4 to 5.4 Mb. The GC content of these strains varied from 40% to 47% (Figure 2A). Functional annotations generated by the RAST server [23] identified an average of 4032.3 coding sequences (ranging between 4010 and 4075) and 91.3 RNAs (from 86 to 95) for non-pigmented strains, while for pigmented strains an average of 4395.6 coding sequences (ranging from 3728 and 4734) and 85.6 RNAs (with a range between 78 to 90) were found (Figure 2A). The coding sequences were classified into subsystems (CO109Y: 491, CO133X: 496, CO302Y: 496, CO325X: 470, CO342X: 494, CO348: 498) and organized into 27 categories according to the hierarchical classification of the SEED databank [24]. The information about the assembly and annotation is summarized in Figure 2A.

### 2.2. Genome Mining and Whole-Genome Sequence-Based Phylogeny

We identified a total of 140 biosynthetic gene clusters (BGCs) in the six analyzed genomes by using antiSMASH [25]. Within them, 19 BGCs were detected for each non-pigmented strain (CO109Y, CO133X, and CO302Y), while 16, 37, and 30 were detected for the pigmented strains CO325X, CO3242X, and CO348, respectively (Figure 2A). Among BGCs in non-pigmented strains can be highlighted those that code for siderophores (desferrioxamine B, with 40% of gene sequence similarity with this cluster according to antiSMASH). Two of the three pigmented strains (CO342X and CO348) were shown to have BGCs, among which stood out those that encode for NRPS and PKS hybrids, for example those that encode the alterochromides and the bromoalterochromides. Additionally, several BGCs of unknown function were found in the six genomes (For detailed information about all the BGCs identified in each strain, see supplementary information Table S1 and Figure S5).

**Figure 3:**
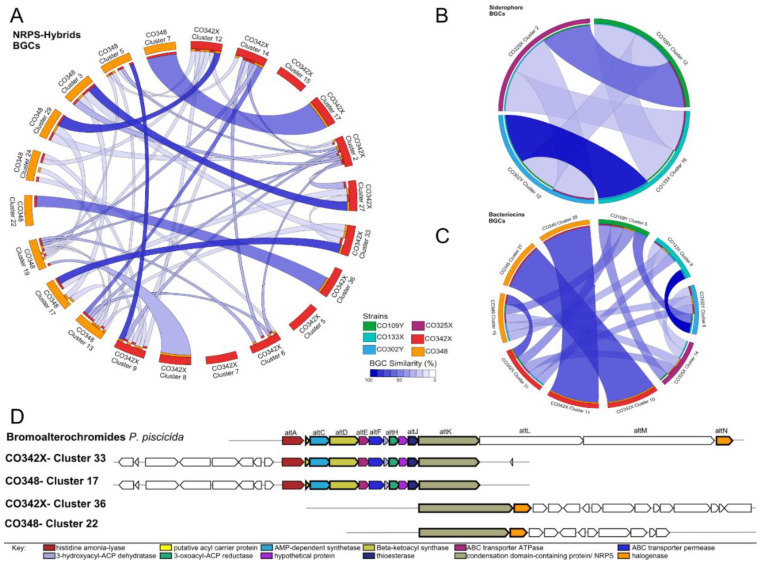
Variation in BGC composition among *Pseudoalteromonas* strains. Overview of the *Pseudoalteromonas* BGC similarity relationships according to MultiGeneBlast analysis by cluster category: (**A**) Nonribosomal peptide synthetases (NRPS)-Hybrids, (**B**) Siderophores, and (**C**) Bacteriocins. The outer track in each figure (**A**–**C**) represents the BGCs analyzed with a different color per strain. The relationships between BGC are shown by colored ribbons indicating their respective level of similarity. (**D**) Synteny map of bromoalterochromide BGC (*P. piscicida* JCM 20779) compared with clusters of pigmented strains CO342X and CO348; all homologous genomic regions were identified by MultiGeneBlast. The most similar BGC is shown immediately below the cluster. The core biosynthetic genes are shown in bold.

To identify the similarity relationships between the identified BGCs, multiple alignments of BGCs were generated using MultiGeneBlast [26] for the categories NRPS-Hybrids, bacteriocins and siderophores (Figure 3). Within the NRPS-Hybrids category, nine BGCs of the strain CO348 and thirteen BGCs of the strain CO342X were aligned. We found that 7 out of 9 BGCs of the strain CO348 are homologous with 7 BCG from CO342X with a BGC similarity above 95% (Figure 2A). Of these, the homologous relationship between CO348-Cluster 17 (ladderane-nrps) and CO342X-Cluster 33 (ladderane-nrps) are distinguished, being that both, according to the results of antiSMASH, have a BGC known for the alterochromides (99% BGC similarity). On the other hand, for CO348-Cluster 22 (NRPS) and CO342X-Cluster 36 (NRPS), it was shown that the core biosynthetic genes of both clusters have a similarity above 98%, the BGC that codes for bromoalterochromides turned out to be the most similar known cluster according to antiSMASH (Figure 3A,D). These four clusters were compared with the characterized biosynthetic gene cluster of bromoalterochromides from *Pseudoalteromanas piscicida* JCM 20,779 (MIBiG: BGC0000314), and we identified homologous genes involved in the biosynthesis of this compound in the sequenced strains (Figure 3D) suggesting that the biosynthetic machinery for bromoalterochromides is present in CO342X and CO348 like in *P. piscicida* JCM 20779. The BGCs CO342X-Cluster 33 and CO348-Cluster 17 contain almost all the biosynthetic components needed to produce the bromoaltherochromides, except for the halogenase gene. While in CO342X-Cluster 26 and CO348-Cluster 22 the halogenase gene is present in the BGC. In the NRPS-Hybrids category (Figure 3A), there is a small proportion of BGCs with high similarity, this suggests that there is a substantial variation in the composition of BGCs among of strains.

Biosynthetic gene clusters of siderophores identified by antiSMASH were also aligned with MultiGeneBlast in order to see homologies between them. A total of four BGCs were identified as siderophores CO109Y-Cluster 12, CO133X-Cluster 16, CO302Y-Cluster 12 and CO325X-Cluster 2 (Figure 2, Table S1). After homology analysis, CO133X and CO302Y displayed a strong relationship between them with a similarity score of 100%. On the other hand, clusters from CO109Y and CO325X are related; however, their similarity value was above 70% (Figure 3B).

A total of 10 BGCs corresponding to bacteriocins were identified in the six analyzed genomes (Figure 2C, Table S1). At least one cluster was identified in the following non-pigmented strains CO109Y (Cluster 5), CO133X (Cluster 8) and CO302Y (Cluster 8). While pigmented strains showed a greater number of bacteriocin clusters (in total 7 BGC), CO325X (Cluster 14), CO342X (Cluster 10, Cluster 11, Cluster 31) and CO348 (Cluster 15, Cluster 27, Cluster 28). Regarding similarity relationship analysis by MultigeneBlast, BGCs that code for bacteriocins in non-pigmented strains have a close relationship between them (Figure 3C), while clusters CO342X-Cluster 31 and CO348-Cluster 15, are the only homologous cluster to CO109Y-Cluster 5, the other clusters from CO342X and CO348 showed to be homologous between them. Interestingly, the cluster 14 that belonged to strain CO325X showed a weak relationship with BGC from both groups (Figure 3C).

A whole Genome Blast Distance Phylogeny (GBDP) was constructed for phylogenetic inference of the studied strains (Figure S6, Table S7) [27]. The *Pseudoalteromonas* strains from Panama were assigned to 4 of 31 clusters of species represented by type strains. According to the GBDP, pigmented strains CO342X and CO348 are strongly related to *Pseudoalteromonas maricaloris* LMG 19692^T^ and *Pseudoalteromonas piscicida* ATCC 15057^T^ with a pseudo-bootstrap branch support of 100% for the cluster, and the other pigmented strain, CO325X, is closely related to *Psedoalteromonas ruthenica* LMG 19699^T^. Non-pigmented strains CO302Y and CO133X clustered together and are closely related to *Pseudoalteromonas arabiensis* JCM 17292^T^, while strain CO109Y is related to *P. shioyasakiensis* JCM 18891^T^.

### 2.3. MS/MS Molecular Networking

**Figure 4:**
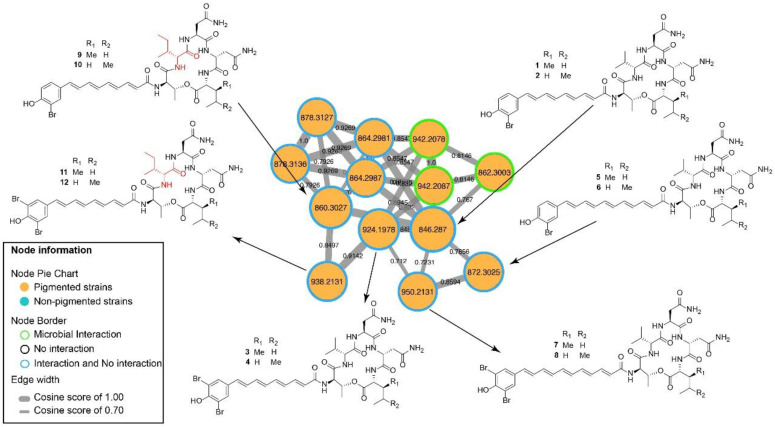
Molecular networking of bromoalterochromide analogs. Arrow indicates the structure of each molecule for compounds **1–8**, and in red the new isoleucine amino acid position for compound **9–12**.

Analysis of the MS/MS data led to the detection of 1966 molecules, which were visualized as nodes in the molecular network. A large number of specialized metabolites, between 8.22 × 10^3^–1.12 × 10^8^ feature intensity sum, were produced by both the bacterial monoculture (103) and bacterial interactions ((600), shared production (931) and control (332). On average, in the non-pigmented strains, between 1.0 × 10^3^–5.94 × 10^6^ feature intensity sum, were identified 284 molecular features while for the pigmented strains a number of 372 molecular features were detected, shared features (978) and control (332); raw network is available at https://gnps.ucsd.edu/ProteoSAFe/status.jsp?task=f1032d7a75504d128e64363eb433efe4. The network was dereplicated using the Global Natural Products Social Molecular Networking (GNPS) [19], which led to the identification of two molecular families including bromoalterochromides and siderophores, among several unknown families. The bromoalterochromides molecular family comprises two clusters assigned to the protonated (Figure 4) and sodiated adducts https://gnps.ucsd.edu/ProteoSAFe/result.jsp?view=network_displayer&componentindex=5&task=f1032d7a75504d128e64363eb433efe4&show=true. Protonated adducts were assigned on the basis of the analog search using the in silico peptidic natural product dereplicator (version 1.2.8) [28], and the manual curation of hits to avoid false positives following standard procedures [29,30,31,32,33], the raw dereplicator result is available at https://gnps.ucsd.edu/ProteoSAFe/status.jsp?task=07191ae6bd5d4121a478ce2572e62ec9. Compounds dereplicated include bromoalterochromide A/A’ (**1**, **2**), dibromoalterochromide A/A’ (**3**, **4**), bromoalterochromide B/B’ (**5–6**) and dibromoalterochromide B/B’ (**7–8**) [34,35] produced by pigmented bacteria (Figure 4); and the siderophore bisucaberin (**13**) [36] was also found in the molecular network associated to non-pigmented bacteria.

## 3. Discussion

The genus *Pseudoalteromas* is well known for its capacity to produce a diversity of bioactive secondary metabolites. The genomes of six strains of *Pseudoalteromonas* collected in Panama, at Coiba National Park in the Pacific Ocean, were sequenced and mined for the production of secondary metabolites. Organic extracts obtained from these strains were analyzed using MS/MS molecular networking revealing the presence of two molecular families including siderophores and bromoalterochromides.

Molecular networking shows ions (compounds) as nodes (circles) that are grouped in clusters according to their MS^2^ spectral similarity (Figure 4). The nodes are connected by edges, where the thicker the edge the more similar the compounds, therefore compounds that cluster together belong to the same structural class or molecular family [19].

The molecular family composed by bromoalterochromides was of considerable extent and importance in the molecular network of *Pseudoalteromonas* (Figure 4). Known members of the family including bromoalterochromides A/A’ (**1**, **2**), bromoalterochromides B/B’ (**5**, **6**) and their dibrominated analogues (**3**, **4**, **7**, **8**) [10,34,35] were present in the network and were dereplicated using GNPS. The structures of compounds **1**–**8** were confirmed by manual annotation of their MS^2^ spectra (Table S6, Figures S3 and S4). Additionally, we found two nodes in the bromoalterochromides cluster, *m/z* 860.3027 and 938.2117, (Figure 4) that could not be assigned to known compounds, hence we proceeded to a more detailed analyses of these metabolites through manual annotation of their MS^2^ spectra finding out they were new compounds named bromoalterochromides D/D’ (**9, 10**), and their dibrominated analogues (**11**, **12**) (Figure 1 and Figure 4).

The molecular network of bromoalterochromides is composed by a single cluster (Figure 4). The cluster includes the bromoalterochromide A series, which possesses an aryl polyene side chain of 15 carbon atoms, and the bromoalterochromide B series with an aryl polyene moiety of 17 carbon atoms that include an additional double bond (Figure 4). The new dibromoalterochromides (**11**–**12**) are part of bromoalterochromide A series, with an aryl polyene moiety of 15 carbon atoms.

**Figure 5:**
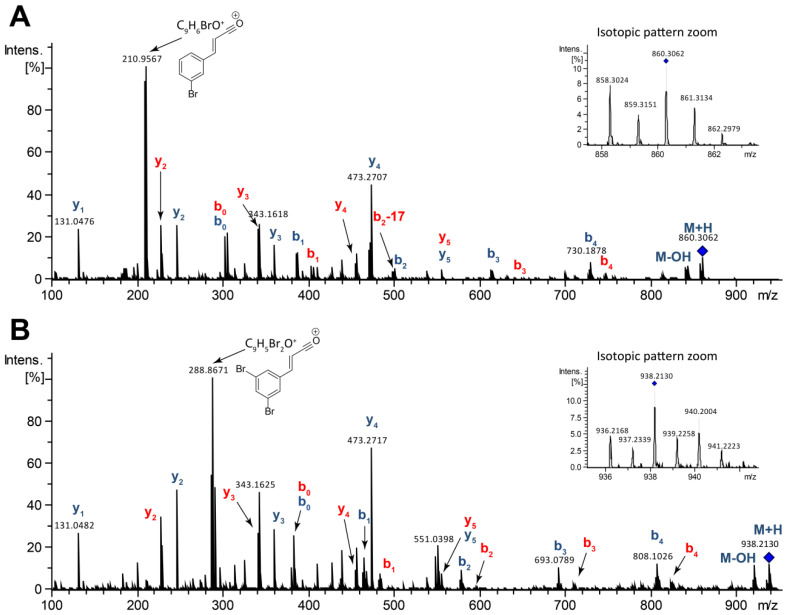
MS/MS spectra annotated for: (**A**) bromoalterochromides D/D’ (**9–10**) and (**B**) dibromoalterochromides D/D’ (**11–12**).

The molecular formula of compound **9** was determined as C_39_H_52_N_7_O_10_Br based on its HR-ESITOFMS data which showed a protonated molecular ion at *m/z* 858.3024 [M + H]^+^ (calcd for C_39_H_53_N_7_O_10_Br, 858.3032). The molecular ion showed an isotopic pattern consistent with a mono-brominated molecule (Figure 5A).

**Figure 6:**
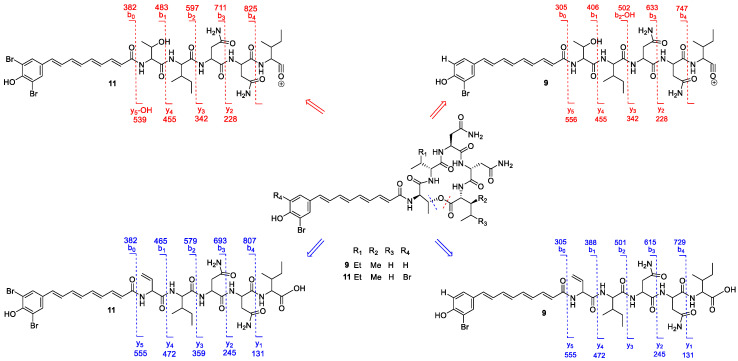
MS^2^ fragments observed for compounds **9** and **11** according to the opening site of the cycle.

MS^2^ spectrum of compound **9** (Figure 5A) indicated the pentapeptide cycle opens at two positions: (i) at the ester bond of the threonine and the C-terminal carbonyl of the isoleucine/leucine residue; and (ii) at the oxygen/carbon bond of the threonine residue (Figure 6), generating a series of protonated *b* and *y* fragments consistent with the sequence of Thr-Ile-Asn-Asn-Ile/Leu (Figure 6). This kind of cycle-opening and fragmentation is in agreement with what has been previously reported for other bromoalterochromides [35].

Most of the peaks in the MS^2^ spectra (Figure 5A) were assigned to the *b* and *y* fragment series, according to the opening of the pentapeptide cycle (Figure 6). The base peak (*m/z* 210.9567) corresponded to the product of the aromatic elimination of the aryl polyene residue present in compound **9** [37]. The mechanism of this elimination reaction is shown in Figure S1.

In the supplementary material published by Ross and collaborators [38] the sodiated adduct of compound **9** was detected in *Pseudoalteromonas piscicida*. Although the structure of the compound was not determined, they indicated it was an analog of bromoalterochromide A plus a CH_2_ group. Recently, while we were finalizing the preparation of this manuscript Suria et al., reported the structures of compounds **9** and **10** characterized by HRMS and NMR [39] from a strain of *Pseudoalteromonas* sp. JC28, however they didn’t find the dibrominated analogues, compounds **11–12,** we are reporting here.

The molecular formula of compound **11** was determined as C_39_H_51_N_7_O_10_Br_2_ based on the HR-ESITOFMS data which showed a protonated molecular ion at *m/z* 936.2168 [M + H]^+^, (calcd for C_39_H_52_N_7_O_10_Br_2_, 936.2137). The molecular ion showed an isotopic pattern consistent with a di-brominated molecule (Figure 5B). Compound **11** showed a MS^2^ spectrum following the same fragmentation pattern and amino acid sequence of compound **9**, and fragments belonging to the *b* and *y* series were the more abundant (Figure 5B and Figure 6). Compounds **11–12** were the dibrominated analogs of compounds **9–10**.

The MS^2^-based identification of the new compounds (**11–12**) is considered level two according to the metabolomics standards initiative (MSI) [40]. Bromoalterochromides D/D’ (**9**, **10**), and their dibrominated analogues (**11**, **12**), respectively, are similar to bromoalaterochromides A/A’ (**1**, **2**), and dibromoalterochromides A/A’ (**3**, **4**). The main difference consists in compounds **9–12** incorporate an amino acid substitution of isoleucine instead the valine residue present in bromoalaterochromides A/A’ and dibromoalterochromides A/A’ (Figure 4). This kind of substitution has been also observed in bromoalterochromides A’’ and B’’ where the C-terminal isoleucine/leucine residue in bromoalterochromide A/A’ was substituted by a valine residue [38]. Valine, leucine and isoleucine amino acid substitutions have been described to occur in microorganisms since their biosynthetic pathways are interrelated [39,41,42] and it is a common strategy used in pharmacology to improve compound stability and activity [43].

Compounds **9–12** were produced by pigmented *Pseudoalteromonas* strains CO348 and CO342X during interactions against *C. albicans*, *A. fumigatus*, *S. aureus* and *E. coli* (Figure 4).

Being that almost all the metabolites that are part of this family were produced with more intensity during microbial interactions. For instance, the known bromoalterochromides (**1–8**) were detected in the interaction between the strains CO348 against *C. albicans*. Similarly, the new bromoalterochromide D series (**9–12**) were also detected during interaction of CO348 and CO342X against *C. albicans*, *A. fumigatus*, *S. aureus*, and *E. coli*. These findings suggest that *Pseudoalteromonas* compounds could provide a defensive barrier against pathogens which will be useful for the host [6,7,8,40,44].

To our knowledge, this is the first report of compounds **11–12**. These compounds are analogs of dibromoalterochromide A/A’ (**3, 4**) [34,35]. According to our antiSMASH analyses, these metabolites are synthesized by NRPS, given that four BGCs from the producers’ strains showed homology to the characterized biosynthetic gene cluster of bromoalterochromides in *P. piscicida* JCM 20,779 (MIBiG: BGC0000314) (Figure 3D).

The siderophore bisucaberin (**13**) was found in the molecular network of non-pigmented *Pseudoalteromonas* strains only. It was observed mainly during the interaction of CO133X and CO302Y against *C. albicans* and *A. fumigatus* (Figure S2). Bisucaberin (**13**) belongs to the family of dihydroxamic acid macrocyclic siderophores used by microbes to acquire iron from the environment. This compound has been reported to have mild activity against *Vibrio* sp. [45] and against cancer cells [46]. The detection of siderophores during interactions against fungi and yeast, suggest that they might have antifungal activity. The BGCs identified as siderophores in our strains were compared against bisucaberin B BGC from *Tenacibaculum mesophilum* (MIBiG: BGC0001531), showing homologous relationships to genes involved in the biosynthesis of this molecule (Figure S2C).

Our results indicate that all the *Pseudoalteromonas* strains studied herein harbor at least one cluster involved in the biosynthesis of bioactive compounds, some of them are reported for the first time for this genus, specifically the hybrid BGCs NRPS involved in the biosynthesis of the new compounds **11–12**.

Nonribosomal peptide synthetases (NRPS) and polyketide synthases (PKS) are multi-enzymatic, multi-domain synthases involved in the biosynthesis of nonribosomal peptides and polyketides. These secondary metabolites display a wide range of biological activities such as antimicrobial, antifungal, antiparasitic, antitumor and immunosuppressive [47]. According to our results, some strains reached a considerable number of distinct NRPS-PKS clusters in the pigmented group. While non-pigmented strains contained clusters of siderophores, resorcinols, and bacteriocins.

The presence of NRPS and PKS BGCs agrees with what has been previously observed in pigmented *Pseudoalteromonas* such as *P. luteoviolaceae* and *P. piscicida* JCM 20779, in this latter the presence of BGCs that code for alterochromides and bromoaltherochromides have also been reported [4,38]. The well-known bromoaltherochromides A/A’ (identified by GNPS), were also observed within the molecular network being produced in monoculture and also during interaction of the pigmented strains CO348 and CO342X against *C. albicans*, *A. fumigatus*, *S. aureus*, and *E. coli*.

Bromoaltherochromides have been reported to be produced by several pigmented strains of *Pseudoalteromonas*, for instance *P. maricaloris* KMM 636^T^ isolated from the sponge *Fascaplysinopsis reticulata* collected at the Great Barrier Reef, where bromoalterochromides A/A’ displayed antimicrobial activity against *B. subtilis*, *S. aureus*, *Enterococcus faecium*, and *C. albicans* [48]. Also, these compounds were isolated from *P. rubra* and *P. flavipulchra* exhibiting antibacterial activity against *Vibrio anguillarum* [45]. Also, our group reported bromoalterochromides A/A’ found in *Pseudoalteromonas* sp. OT59 isolated from the octocoral *Leptogorgia alba* collected at Otoque Island (Pacific coast, Panama) showing antifungal and antibacterial activity against *Penicillium citrinum* and *B. subtilis*, respectively [9,10].

In general, bromoaltherochromides are compounds produced by a range of pigmented *Pseudoalteromonas* and the evidence suggests that these compounds are part of the defense mechanism used by their hosts against microbial pathogens. Here we found several analogs of these compounds (**1–12**) including the new dibromoalterochromides D/D’ (**11–12**). Importantly they are produced by strains that represent putative new species of *Pseudoalteromonas* [12] which were found in association with the octocorals *Muricea* sp. and *Leptogorgia cofrini* from Coiba National Park (Panama).

Our results show that four of the six strains sequenced (CO109Y, CO133X, CO302Y, and CO325X) have genes encoding for siderophores, an important tool in microbial competition in the marine environment due to the low concentrations of bioavailable iron in seawater [4,49,50]. The identification of siderophores in *Pseudoalteromonas* has been previously reported in *P. agarivorans* S816 and *P. ruthenica* S3258 [4]. Furthermore, in *Pseudoalteromonas* sp. KP20–4, the siderophores pseudoalterobactin A and B were identified [50]. Here our molecular networking analyses showed that the siderophore bisucaberin is produced by the non-pigmented *Pseudoalteromonas* strains CO133X and CO302Y (from octororals *Pacifigorgia smithsoniana* and *Psammogorgia* sp., respectively), some of them during interaction against *C. albicans* and *A. fumigatus*. This molecule was reported in *Vibrio salmonicida*, and *P. haloplanktis* for its activity against tumoral cells [51].

The number of gene clusters identified as bacteriocins in this study varied between one and three in each strain. Bacteriocins are proteinaceous antibacterial compounds that are produced mainly by bacteria; they are ribosomally synthesized and exhibit a narrow spectrum of bioactivity. Different bacteriocins capable of inhibiting Gram-positive and Gram-negative bacteria have been reported to be produced by *Pseudoalteromonas* spp. Examples include AlpP (L-Lysine oxidase) from *P. tunicata* D2, L-amino acid oxidase from *P. luteoviolacea*, CPMOR-1 and the compound PfaP from *P. flavipulchra* JG1 [7].

The pigmented strains sequenced here are characterized by the presence of high number of BGCs, as has been previously found by Bosi et al. for other species of *Pseudoalteromonas* [5], where pigmented species (*P. citrea*, *P. rubra*, *P. piscicida* and *P. flavipulchra*) presented a higher number of BGCs, particularly for the PKS and NRPS categories. Additionally, in our study bacteriocin clusters were shared by all *Pseudoalteromonas* strains (pigmented and non-pigmented), while other categories like PKS and NRPS were only present in the pigmented group. These findings and the results presented here suggest a gain/lost scenario of these genes during their evolutionary history, specifically in pigmented strains [5].

Despite the case of an increase in the number of BGCs and the bioactivity of pigmented strains there are cases where the bioactivity cannot be predicted by genome mining. For example, the strain CO325X (from the octocoral *Muricea austerea*), closely related to *P. ruthenica* according 16s rRNA gene, presented a highly antagonistic activity against *B. subtillis*, *B. pumilus*, *S. aureus*, *A. baumanii*, *C. albicans*, and *A. fumigatus*, in agar-based screening assays [12]. We identified 16 clusters related to secondary metabolism, and most of them are similar to the ones identified in non-pigmented strains and also in *P. ruthenica* according the antiSMASH database (Table S3). Several molecules, some of them peptides detected by MS/MS molecular networking, possibly involved in its antimicrobial activity, were not detected by genome mining. This could be due to limitations in the prediction algorithms of biosynthetic gene clusters that may be involved.

*Pseudoalteromonas* from Panama represent a source of highly bioactive molecules that need to be further explored. Clearly, the integration of genome mining and detection of microbial metabolites by molecular networking are complementary techniques to the biological screenings of molecules. This combination of genome mining, molecular networking, and antimicrobial assays allowed the discovery of the new dibromoalterochromides D/D’ (**11–12**); and new sources of siderophores, resorcinols and bacteriocins. This approach promises to be valuable in the discovery of new drugs from marine sources.

## 4. Materials and Methods

### 4.1. Bacterial Strains and Growth Conditions

Species of *Pseudoalteromonas* were isolated from octocorals collected at Coiba National Park, Panama, in 2009. These strains were selected based on their antimicrobial activity against Gram-positive and Gram-negative bacteria as well as antifungal activity against *Aspergillus fumigatus* and *Candida albicans* (Table 1). All isolates were initially identified as *Pseudoalteromonas* spp. based on the sequence analysis of 16S rRNA gene [12]. The strains used in this study were grown on the M1 agar medium Agar/Broth at room temperature. Pure cultures of each strain were stored at −80 ℃ in a cryoprotectant solution until this study.

### 4.2. Genomic DNA Isolation and Sequencing

Genomic DNA was extracted using Gentra Puregene Yeasts/Bact. Kit (QIAGEN, Hilden, Germany) following the DNA purification protocol for Gram-Negative Bacteria. DNA concentration was measured by using a NanoDrop 2000 (Thermo Fisher Scientific, Waltham, MA, USA) and Qubit analyzer (ThermoFisher Scientific, Waltham, MA, USA). Also, DNA condition was revealed by a 1% agarose gel electrophoresis. Genome sequencing was performed by Macrogen Inc. (Seoul, Korea). Library of 350 bp (Illumina TruSeq DNA PCR-Free) were used for 100 bp paired-end sequencing genomes using Illumina sequencing technology HiSeq2000 (Illumina, San Diego, CA, USA).

### 4.3. Genome Assembly and Annotation

De novo genome assembly was performed using SPAdes assembler 3.11.1 [52] (St. Petersburg State University, Russia) with a coverage of 75× or higher. Genome assemblies were evaluated using QUAST. The draft genomes were annotated by RAST server (Rapid Annotation using Subsystem Technology) [22] using RAST as a gene caller. Sequences shorter than 200 nucleotides were trimmed from all draft genomes as a requirement for Genbank submission. All of the draft genomes were submitted to the National Center for Biotechnology Information (NCBI) database under the accession numbers: SGPD00000000 (CO109Y), SGPE00000000 (CO133X), SGPF00000000 (CO302Y), SGPG00000000 (CO325X), SGPH00000000 (CO342X), and SGPI00000000 (CO348).

### 4.4. Genome Mining and Whole-Genome Sequence-Based Phylogeny

Biosynthetic gene clusters were predicted by using the genome mining tool AntiSMASH v3.0 using the default parameters and incorporation of the ClusterFinder algorithm [24]. To visualize the BGCs categories predicted by AntiSMASH in *Pseudoalteromonas* strains genomes, a heat map was performed through R packages ggplot2 [53] and reshape [54].

To visualize similarities among BGCs in *Pseudoalteromonas*, the MultiGeneBlast algorithm was used [25] where AntiSMASH cluster output files were used as input to perform the similarity analysis, each output file was modified slightly to contain the strain name and cluster number to make results easier to interpret. The MultiGene Blast database used for the analysis included the categories NRPS-hybrids, bacteriocins and siderophores. Predicted clusters were blasted one by one against these databases. Default settings were used when running MultiGeneBlast.

A Genome BLAST Distance Phylogeny (GBDP) approach was used to infer evolutionary relationships of the studied strains with type strains of *Pseudoalteromonas*. The genome sequence data were uploaded to the Type (Strain) Genome Server (TYGS), a bioinformatics platform available at https://tygs.dsmz.de, for a whole genome-based taxonomic analysis [26]. In brief, the TYGS analysis was performed as follow: determination of closest type strain genomes was done by a comparison of the strains sequenced in this study against all type strain genomes available in the TYGS database via the MASH algorithm, a fast approximation of intergenomic relatedness [55]. This was used as a proxy to find the best 50 matching type strains for each *Pseudoalteromonas* strain genome and to subsequently calculate precise distances using the GBDP approach, under the algorithm “coverage” and distance formula d5 [56]. These distances were finally used to determine the 10 closest type strain genomes for each sample. The resulting intergenomic distances were used to infer a balanced minimum evolution tree via FASTME 2.1.4 including SPR postprocessing [57]. Branch support was inferred from 100 pseudo-bootstrap replicates each and visualized with PhyD3 [58].

### 4.5. Crude Extracts Preparation for LC-MS/MS Analysis

Antimicrobial interactions were performed to obtain crude extract of *Pseudoalteromonas* strains selected for whole genome sequencing (Table 1). Suspensions of *Staphylococcus aureus* ATCC 43300, *Escherichia coli* ATCC 10,536 and *Candida albicans* ATCC 10,231 were spread on petri dishes containing M1 agar at 0.5 McFarland (1.5 × 10^8^ CFU/mL), while for *Aspergillus fumigatus* ATCC 1028 a conidial solution was prepared at an optical density of 0.09–0.11 (0.6–5 × 10^6^ CFU/mL). After spreading all testers on M1 plates, 21 cumulus of pure culture of *Pseudoalteromonas* strains were inoculated (See Table 1 for interaction details). All plates were incubated at 30 °C for 24 to 72 h. The inhibition halo was cut, put into an Erlenmeyer flask of 500 mL and macerated with 100 mL of ethyl acetate and gently shaken for 24 h at room temperature. Ethyl acetate was filtered and concentrated by rotary evaporation, dissolved in Methanol, and concentrated.

### 4.6. LC-MS/MS Analysis

Organic extracts (0.05 mg) were resuspended in LC-MS grade 80% MeOH/Water containing 2µM sulfamethazine as internal standard. LC-MS/MS analysis was performed in an UltiMate 3000 UPLC system (ThermoFisher Scientific, Waltham, MA, USA) using a Scherzo SM-C18 (Imtakt USA, Portland, OR, USA) column (250 × 2 mm, 3 µm) and Maxis Q-TOF mass spectrometer (Bruker Daltonics, Billerica, MA, USA) equipped with ESI source. Isocratic elution with 100% solvent A (LC-MS grade 99.9% water, 0.1% formic acid) for 5 min, followed by a linear gradient from 100% A to 100% B (LC-MS grade 99.9% acetonitrile, 0.1% formic acid) in 5 min, held at 100% B for 2 min. Then, 100% B to 100% A in 2.5 min and maintained at 100% A for 1 min, linear gradient from 100% A to 100% B in 2 min, held at 100% B for 1 min, 100% B to 100% A in 1 min and held at 100% A for 1.5 min. A flow rate of 0.5 mL/min throughout the 21 min run was maintained. MS spectra were acquired in positive ion mode in the range of 100–2000 *m/z.* A mixture of 10 mg/mL of each sulfamethazine, sulfamethizole, sulfachloropyridazine, sulfadimethoxine, amitriptyline, and coumarin was run after every 96 injections for quality control. An external calibration with ESI-Low concentration tuning mix (Agilent technologies) was performed prior to data collection and internal calibrant Hexakis (1H,1H,2H-perfluoroethoxy) phosphazene (CAS 186817-57-2) was used throughout the runs. The capillary voltage of 4500 V, nebulizer gas pressure (nitrogen) of 2 bar, ion source temperature of 200 °C, dry gas flow of 9 L/min source temperature, spectral rate of 3 Hz for MS^1^ and 10 Hz for MS^2^ was used. For acquiring MS/MS fragmentation, 5 of the most intense ions per MS^1^ were selected. Advanced stepping function used to fragment ions and collision-induced dissociation (CID) energies for MS/MS data acquisition are presented in supplementary information (Tables S4 and S5).

MS/MS active exclusion parameter was set to 2 and released after 30 s. The mass of internal standard was excluded from the MS/MS list using a mass range of *m*/*z* 621.5–623.0. The data were deposited in the MassIVE online repository (MSV000083295).

### 4.7. MZmine Preprocessing Workflow for Molecular Networking

Extracts from *Pseudoalteromonas* strains monocultures, and interactions against *C. albicans*, *A. fumigatus*, *S. aureus*, and *E. coli* were used to facilitate the identification of the metabolites observed during their growth (Figure 4, Table 1). After LC/MS experiments, MS/MS data were exported to 32-bit mzXML file, using Bruker Compass Data analysis v4.1(Bruker Daltonics, Billerica, MA USA). These files were imported to MZmine 2.37.1 for feature detection [59]. Feature extraction was performed for centroid mass detector with a signal threshold of 5.0 × 10^3^ for MS^1^ and 5.0 × 10^2^ for MS^2^. Chromatogram builder was run with a minimum height of 5.0 × 10^3^ and tolerance of 10 ppm. This step connects all consecutive *m/z* values over multiple scans and converts them into chromatograms. Then, the chromatograms were deconvoluted with a peak duration range of 0.01 to 1.00 min and a baseline cut-off algorithm of 2.0 × 10^3^. Additionally, isotopic peaks were grouped with a *m*/*z* tolerance of 0.005 Da and a retention time of 0.20 min. This step was applied in order to find peaks forming an isotopic pattern. Within this isotopic pattern, the most intensive peaks were considered for being assembled within a single feature. As multiple files were processed, retention time alignment step was required for matching relevant peaks across multiple peak lists. Thus, relevant detected peaks were aligned through Join Aligner Module considering 0.02 Da and retention time tolerance of 0.2 min. MGF file generated from MZmine 2.33 was uploaded to the Global Natural Products Social Molecular Networking online platform (GNPS) for generating a feature-based molecular network (https://ccms-ucsd.github.io/GNPSDocumentation/featurebasedmolecularnetworking/) [19,60]. This molecular network was generated by filtering edges to have a cosine score above 0.70 and more than 4 matched peaks. The spectra in the network were then searched against GNPS public spectral libraries. The network and parameters can be accessed at the following link (https://gnps.ucsd.edu/ProteoSAFe/status.jsp?task=f1032d7a75504d128e64363eb433efe4) and https://gnps.ucsd.edu/ProteoSAFe/status.jsp?task=07191ae6bd5d4121a478ce2572e62ec9. This network was consequently imported to Cytoscape version 3.8.0 (www.cytoscape.org) for visualization and analysis [61]. Group mapping was created by R language in Jupyter Notebook (http://jupyter.org/) through Anaconda Navigator.

### 4.8. MS/MS Spectrum Curation and Annotation

Network clusters that contained molecules of interest were curated by manual annotation of each compound following standard procedures [29,30,31,32,33] and compared to fragmentation patterns previously reported for other bromoalterochromides [35]. Briefly, raw spectra were analyzed using Bruker Compass Data Analysis 4.1 SR1, each node parent mass within the cluster was used to generate an extract ion chromatogram; then monoisotopic mass was used to calculate ion formula using Bruker Smart Formula (Bruker Daltonics, Billerica, MA, USA) manually. Theoretical fragmentation was generated using ChemBioDraw 15.1.0.144 (PerkinElmer, Waltham, MA, USA) and the annotation was performed by comparing theoretical scheme with the MS/MS spectra following standard procedures [29,30,31,32,33].

## 5. Conclusions

Our results confirm the genetic potential of octocoral-associated *Pseudoalteromonas* as a promising source of natural products with antimicrobial activity. Strains studied herein, some of which are putative new species of the genus [10], possess BGCs for the production of secondary metabolites, and a considerable number of distinct PKS and NRPS clusters were found in pigmented strains, where substantial variation in the BCGs composition was found. The integration of genome mining, MS/MS molecular networking and in vitro microbial interactions constitute powerful tools for detecting and functionally annotate novel compounds of diverse biological activities. The application of this approach led to the identification of the new dibromoalterochromide D/D’ (**11–12**); in addition to new sources of siderophores and bacteriocins.

## Figures and Tables

**Table 1:** *Pseudoalteromonas* strains under study and their antimicrobial activity.

Strain	Host Species *	Collection Site	Pigmentation	Antimicrobial Activity ** Microbial Species Inhibited and Diameter of Inhibition Halo
CO109Y	*Eugorgia daniana*	Coiba National Park: Roca Hacha	Non-pigmented	***Candida albicans *** (14.0 mm)
CO133X	*Pacifigorgia smithsoniana*	Coiba National Park: Catedral	Non-pigmented	***C. albicans*** (11.0 mm) ***Aspergillus fumigatus*** (11.0 mm)
CO302Y	*Psammogorgia* sp.	Coiba National Park: Roca Hacha	Non-pigmented	***A. fumigatus *** (9.5 mm)
CO325X	*Muricea austera*	Coiba National Park: Roca Hacha	Pigmented (light yellow)	*Bacillus subtillis* (20.0 mm) *B. pumillus* (8.0 mm) ***Staphylococcus aureus*** (29.0 mm) *Acinetobacter baumanii* (11.0 mm) ***C. albicans*** (28.0 mm) ***A. fumigatus*** (21.0 mm)
CO342X	*Muricea* sp.	Coiba National Park: Roca Hacha	Pigmented (Yellow)	*B. subtillis* (9.0 mm) *B. pumillus* (7.0 mm) ***S. aureus ***(16.0 mm) *P. haloplanktis* (8.5 mm)
CO348	*Leptogorgia cofrini*	Coiba National Park: Catedral	Pigmented (Yellow)	*B. subtillis* (14.0 mm) *B. pumillus* (8.5 mm) ***S. aureus*** (30.5 mm) *Vibrio coralliilyticus* (9.0 mm) *P. haloplanktis* (12.0 mm) *A. baumanii* (9.0 mm) *Pseudomonas aeruginosa* (14.0 mm) ***Escherichia coli*** (15.0 mm) ***C. albicans*** (26.0 mm) ***A. fumigatus ***(18.0 mm)

* All host species are octocorals. ** Antimicrobial interactions used for organic extractions are in bold and underlined.

## Supplementary Materials

The following are available online at https://www.mdpi.com/1660-3397/18/9/456/s1, Table S1: Identified BGC in Pseudoalteromonas by antiSMASH, Table S2: MultiGeneBlast results per category, Table S3: Summary of BGCs found in *Pseudoalteromonas* species according to antiSMASH database, Table S4: MS1 peaks considered for MS2 fragmentation data, Table S5: Collision-induced dissociation (CID) energies for MS/MS data acquisition, Table S6: Observed CID fragment ions for characterized bromoalterochromides, Table S7. Pairwise comparisons of *Pseudoalteromonas* strains genomes vs. type strain genomes in Type (Strain) Genome Server (TSGS), Figure S1: Aromatic elimination of aryl polyenes, Figure S2: Bisucaberin, Figure S3: MS/MS spectra for compounds **1–4**. Figure S4: MS/MS spectra for compounds **5–8**, Figure S5. Heatmap containing BGCs categories predicted by AntiSMASH in *Pseudoalteromonas* strains genomes, Figure S6. Whole-genome sequence-based Genome Blast Distance Phylogeny (GBDP) tree of *Pseudoalteromonas* strains from Coiba.
