# Supplementary material for: Genome Mining, Microbial Interactions, and Molecular Networking Reveals New Dibromoalterochromides from Strains of Pseudoalteromonas of Coiba National Park-Panama
Source: Mar Drugs. 2020 Sep 3;18(9):456. doi: 10.3390/md18090456 (PMC7551054; doi:10.3390/md18090456)
Supplement: Supplementary file 1 [file marinedrugs-18-00456-s001.pdf]

# Genome mining, microbial interactions and molecular networking reveals new dibromoalterochromides from strains of *Pseudoalteromonas* of Coiba National Park- Panama.

Librada A. Atencio[1,2], Cristopher A. Boya P.[1,2], Christian Martin H.[1,2], Luis C. Mejía[1,3,*], Pieter C. Dorrestein[4], and Marcelino Gutiérrez[1,*]

[1] Centro de Biodiversidad y Descubrimiento de Drogas, Instituto de Investigaciones Científicas y Servicios de Alta Tecnología (INDICASAT), Clayton, Panama; latencio@indicasat.org.pa (L.A.A), christian.martin.hdz@gmail.com (C.M.), CBoya@indicasat.org.pa (C.A.B.P.), LMejia@indicasat.org.pa (L.C.M.).
[2] Department of Biotechnology, Acharya Nagarjuna University, Nagarjuna Nagar, Guntur 522510, India.
[3] Smithsonian Tropical Research Institute, Balboa, Ancón, Panama.
[4] Collaborative Mass Spectrometry Innovation Center, Skaggs School of Pharmacy and Pharmaceutical Sciences, University of California San Diego, La Jolla, California, USA.
* Correspondence: mgutierrez@indicasat.org.pa; Tel.: +507-517-0732; Fax: +507-517-0701; lmejia@indicasat.org.pa; Tel.: +507-517-0700; Fax: +507-517-0701

Table S1: **Identified BGC in *Pseudoalteromonas* by antiSMASH**
Table S2: **MultiGeneBlast Results per category**
Table S3: **Summary of BGC found in *Pseudoalteromonas* species according to antiSMASH database**
Table S4: **MS1 peaks considered for MS2 fragmentation data**
Table S5: **Collision-induced dissociation (CID) energies for MS/MS data acquisition**
Table S6: **Observed CID fragment ions for characterized bromoalterochromides**
Table S7. **Pairwise comparisons of *Pseudoalteromonas* strains genomes vs type strain genomes in Type (Strain) Genome Server (TSGS)**
Figure S1: **Aromatic elimination of aryl polyenes**
Figure S2: **Bisucaberin**
Figure S3. **MS/MS spectra for bromoalterochromide A/A′ (1,2) and dibromoalterochromide A/A′ (3,4)**
Figure S4. **MS/MS spectra for bromoalterochromide B/B′ (5,6) and dibromoalterochromide B/B′ (7,8)**
Figure S5. **Heatmap containing BGCs categories predicted by AntiSMASH in *Pseudoalteromonas* strains genomes.**
Figure S6. **Genome Blast Distance Phylogeny (GBDP) of *Pseudoalteromonas* strains from Coiba**

**Table S1. Identified BGC in *Pseudoalteromonas* by antiSMASH**

| Cluster | Input accession number | Input name *Pseudoalteromonas* sp. | Gene cluster type | from | to | Most similar known cluster (MIBiG) | Similarity | MIBiG BGC-ID |
|---|---|---|---|---|---|---|---|---|
| 1 | c00002_NODE_7_length_275579_cov_91.798942 | CO109Y | putative | 209438 | 216420 | - | | |
| 2 | c00008_NODE_27_length_28775_cov_95.895672 | CO109Y | putative | 12029 | 20440 | Exopolysaccharide | 13% | BGC0000766_c1 |
| 3 | c00013_NODE_28_length_27831_cov_96.346797 | CO109Y | putative | 8665 | 25857 | Emulsan | 13% | BGC0000760_c1 |
| 4 | c00022_NODE_8_length_245287_cov_75.255230 | CO109Y | fatty acid | 156299 | 177372 | - | | - |
| 5 | c00025_NODE_5_length_301484_cov_74.847426 | CO109Y | bacteriocin | 74499 | 85362 | - | | - |
| 6 | c00025_NODE_5_length_301484_cov_74.847426 | CO109Y | fatty acid | 269827 | 291725 | Xenocyloins | 25% | BGC0000189_c1 |
| 7 | c00028_NODE_2_length_436453_cov_71.787067 | CO109Y | saccharide | 120799 | 160399 | Taxlllaid | 4% | BGC0001133_c1 |
| 8 | c00028_NODE_2_length_436453_cov_71.787067 | CO109Y | putative | 194074 | 198925 | - | | - |
| 9 | c00028_NODE_2_length_436453_cov_71.787067 | CO109Y | putative | 292380 | 299876 | - | | - |
| 10 | c00028_NODE_2_length_436453_cov_71.787067 | CO109Y | putative | 319275 | 326198 | - | | - |
| 11 | c00034_NODE_6_length_297666_cov_83.368656 | CO109Y | putative | 124035 | 140946 | - | | - |
| 12 | c00035_NODE_3_length_421190_cov_82.305811 | CO109Y | siderophore | 98509 | 110908 | Desferrioxamine B | 60% | BGC0000941_c1 |
| 13 | c00035_NODE_3_length_421190_cov_82.305811 | CO109Y | fatty acid | 373851 | 395065 | Alterochromides | 9% | BGC0000299_c1 |
| 14 | c00039_NODE_4_length_315195_cov_104.192787 | CO109Y | putative | 173020 | 188999 | - | | - |
| 15 | c00042_NODE_1_length_523833_cov_79.969463 | CO109Y | putative | 24028 | 39627 | - | | - |
| 16 | c00042_NODE_1_length_523833_cov_79.969463 | CO109Y | putative | 82738 | 88707 | - | | - |
| 17 | c00042_NODE_1_length_523833_cov_79.969463 | CO109Y | arylpolyene | 123335 | 166888 | APE Vf | 45% | BGC0000837_c1 |
| 18 | c00042_NODE_1_length_523833_cov_79.969463 | CO109Y | putative | 222628 | 230409 | - | | - |
| 19 | c00044_NODE_17_length_70298_cov_94.478190 | CO109Y | putative | 49844 | 55847 | - | | - |
| 1 | c00001_NODE_16_length_57928_cov_96.696012 | CO133X | putative | 36924 | 43300 | - | | - |
| 2 | c00002_NODE_6_length_314560_cov_90.082326 | CO133X | fatty acid | 298607 | 314560 | Alterochromides | 9% | BGC0000299_c1 |
| 3 | c00004_NODE_8_length_185114_cov_100.635624 | CO133X | resorcinol | 15829 | 56938 | - | | - |
| 4 | c00004_NODE_8_length_185114_cov_100.635624 | CO133X | putative | 161210 | 174968 | - | | - |

**Table S1. Identified BGC in *Pseudoalteromonas* by antiSMASH (*Continuation*)**

| Cluster | Input accession number | Input name *Pseudoalteromonas* sp. | Gene cluster type | from | to | Most similar known cluster (MIBiG) | Similarity | MIBiG BGC-ID |
|---|---|---|---|---|---|---|---|---|
| 5 | c00005_NODE_4_length_398869_cov_98.406831 | CO133X | putative | 186350 | 199672 | Lipopolysaccharide | 18% | BGC0000773_c1 |
| 6 | c00005_NODE_4_length_398869_cov_98.406831 | CO133X | saccharide | 214996 | 242108 | O-antigen | 10% | BGC0000788_c1 |
| 7 | c00011_NODE_5_length_322967_cov_90.495317 | CO133X | fatty acid | 36504 | 60082 | - | | - |
| 8 | c00017_NODE_14_length_75731_cov_85.392815 | CO133X | bacteriocin | 1 | 6155 | - | | - |
| 9 | c00018_NODE_2_length_617072_cov_80.445482 | CO133X | fatty acid | 151522 | 173420 | Xenocyloins | 25% | BGC0000189_c1 |
| 10 | c00018_NODE_2_length_617072_cov_80.445482 | CO133X | saccharide | 303578 | 343175 | Taxlllaid | 4% | BGC0001133_c1 |
| 11 | c00018_NODE_2_length_617072_cov_80.445482 | CO133X | putative | 374102 | 378942 | - | | |
| 12 | c00018_NODE_2_length_617072_cov_80.445482 | CO133X | putative | 493014 | 511833 | Capsular polysaccharide | 18% | BGC0000729_c1 |
| 13 | c00018_NODE_2_length_617072_cov_80.445482 | CO133X | putative | 519770 | 526669 | - | | - |
| 14 | c00022_NODE_1_length_907290_cov_84.548418 | CO133X | putative | 189606 | 195351 | - | | - |
| 15 | c00022_NODE_1_length_907290_cov_84.548418 | CO133X | fatty acid | 523690 | 544763 | - | | - |
| 16 | c00022_NODE_1_length_907290_cov_84.548418 | CO133X | siderophore | 583940 | 595802 | Desferrioxamine B | 40% | BGC0000941_c1 |
| 17 | c00022_NODE_1_length_907290_cov_84.548418 | CO133X | putative | 705428 | 711382 | - | | - |
| 18 | c00022_NODE_1_length_907290_cov_84.548418 | CO133X | arylpolyene | 749445 | 792998 | APE Vf | 40% | BGC0000837_c1 |
| 19 | c00026_NODE_3_length_446844_cov_94.819338 | CO133X | putative | 227732 | 233664 | - | | - |
| 1 | c00003_NODE_3_length_435531_cov_70.772293 | CO302Y | saccharide | 122027 | 161624 | Taxlllaid | 4% | BGC0001133_c1 |
| 2 | c00003_NODE_3_length_435531_cov_70.772293 | CO302Y | putative | 192551 | 197391 | - | | - |
| 3 | c00003_NODE_3_length_435531_cov_70.772293 | CO302Y | putative | 311463 | 330282 | Capsular polysaccharide | 18% | BGC0000729_c1 |
| 4 | c00003_NODE_3_length_435531_cov_70.772293 | CO302Y | putative | 338219 | 345118 | - | | - |
| 5 | c00008_NODE_17_length_57928_cov_86.112064 | CO302Y | putative | 36924 | 43300 | - | | - |
| 6 | c00009_NODE_4_length_398869_cov_88.401239 | CO302Y | putative | 186350 | 199672 | Lipopolysaccharide | 18% | BGC0000773_c1 |
| 7 | c00009_NODE_4_length_398869_cov_88.401239 | CO302Y | saccharide | 214996 | 242108 | O-antigen | 10% | BGC0000788_c1 |
| 8 | c00016_NODE_15_length_75743_cov_75.636783 | CO302Y | bacteriocin | 1 | 6167 | - | | - |

**Table S1. Identified BGC in *Pseudoalteromonas* by antiSMASH (*Continuation*)**

| Cluster | Input accession number | Input name *Pseudoalteromonas* sp. | Gene cluster type | from | to | Most similar known cluster (MIBiG) | Similarity | MIBiG BGC-ID |
|---|---|---|---|---|---|---|---|---|
| 9 | c00021_NODE_6_length_314546_cov_79.228725 | CO302Y | fatty acid | 298607 | 314546 | Alterochromides | 9% | BGC0000299_c1 |
| 10 | c00022_NODE_1_length_907290_cov_75.576858 | CO302Y | putative | 189606 | 195351 | - | | - |
| 11 | c00022_NODE_1_length_907290_cov_75.576858 | CO302Y | fatty acid | 523690 | 544763 | - | | - |
| 12 | c00022_NODE_1_length_907290_cov_75.576858 | CO302Y | siderophore | 583940 | 595802 | Desferrioxamine B | 40% | BGC0000941_c1 |
| 13 | c00022_NODE_1_length_907290_cov_75.576858 | CO302Y | putative | 705428 | 711382 | - | | - |
| 14 | c00022_NODE_1_length_907290_cov_75.576858 | CO302Y | arylpolyene | 749445 | 792998 | APE Vf | 40% | BGC0000837_c1 |
| 15 | c00023_NODE_8_length_185114_cov_91.426385 | CO302Y | resorcinol | 15829 | 56938 | - | | - |
| 16 | c00023_NODE_8_length_185114_cov_91.426385 | CO302Y | putative | 161210 | 174968 | - | | - |
| 17 | c00024_NODE_5_length_322967_cov_79.767407 | CO302Y | fatty acid | 36504 | 60082 | - | | - |
| 18 | c00026_NODE_2_length_446850_cov_83.984274 | CO302Y | putative | 227738 | 233670 | - | | - |
| 19 | c00029_NODE_9_length_181499_cov_73.333543 | CO302Y | fatty acid | 151545 | 173443 | Xenocyloins | 25% | BGC0000189_c1 |
| 1 | c00003_NODE_10_length_161627_cov_96.675339 | CO325X | fatty acid | 53745 | 74857 | - | | |
| 2 | c00009_NODE_1_length_593470_cov_98.282885 | CO325X | siderophore | 137198 | 149594 | Desferrioxamine B | 60% | BGC0000941_c1 |
| 3 | c00009_NODE_1_length_593470_cov_98.282885 | CO325X | resorcinol | 206269 | 247369 | - | | |
| 4 | c00009_NODE_1_length_593470_cov_98.282885 | CO325X | putative | 275440 | 284485 | - | | |
| 5 | c00009_NODE_1_length_593470_cov_98.282885 | CO325X | putative | 544910 | 551250 | - | | |
| 6 | c00012_NODE_8_length_195902_cov_86.100876 | CO325X | putative | 163725 | 172598 | - | | |
| 7 | c00013_NODE_2_length_386893_cov_96.205092 | CO325X | putative | 204103 | 211799 | - | | |
| 8 | c00013_NODE_2_length_386893_cov_96.205092 | CO325X | putative | 299298 | 307528 | - | | |
| 9 | c00019_NODE_5_length_274675_cov_86.289230 | CO325X | putative | 15853 | 23851 | - | | |
| 10 | c00019_NODE_5_length_274675_cov_86.289230 | CO325X | putative | 205727 | 212760 | - | | |
| 11 | c00022_NODE_3_length_311950_cov_92.901688 | CO325X | fatty acid | 11490 | 32704 | Alterochromides | 9% | BGC0000299_c1 |
| 12 | c00028_NODE_15_length_91940_cov_92.735744 | CO325X | arylpolyene | 1 | 30285 | APE Vf | 40% | BGC0000837_c1 |

**Table S1. Identified BGC in *Pseudoalteromonas* by antiSMASH (*Continuation*)**

| Cluster | Input accession number | Input name *Pseudoalteromonas* sp. | Gene cluster type | from | to | Most similar known cluster (MIBiG) | Similarity | MIBiG BGC-ID |
|---|---|---|---|---|---|---|---|---|
| 13 | c00029_NODE_29_length_18399_cov_109.61 6308 | CO325X | putative | 1320 | 8776 | - | | |
| 14 | c00034_NODE_16_length_88477_cov_89.274 378 | CO325X | bacteriocin | 34655 | 45500 | - | | |
| 15 | c00035_NODE_6_length_236743_cov_103.45 2938 | CO325X | saccharide | 179287 | 220707 | O-antigen | 22% | BGC0000784_c1 |
| 16 | c00036_NODE_11_length_132357_cov_94.48 8917 | CO325X | putative | 32163 | 37566 | - | | |
| 1 | c00002_NODE_22_length_77183_cov_56.043 421 | CO342X | fatty acid | 1 | 21213 | - | | - |
| 2 | c00013_NODE_39_length_6543_cov_70.8266 32 | CO342X | nrps | 1 | 6543 | - | | - |
| 3 | c00014_NODE_13_length_160050_cov_62.43 7136 | CO342X | putative | 44802 | 64448 | - | | - |
| 4 | c00017_NODE_11_length_173480_cov_64.73 8107 | CO342X | putative | 93837 | 96329 | - | | - |
| 5 | c00019_NODE_42_length_4032_cov_72.4884 96 | CO342X | nrps | 1 | 4032 | - | | - |
| 6 | c00020_NODE_21_length_80338_cov_74.548 211.. | CO342X | nrps | 1 | 47394 | Bacillibactin | 60% | BGC0001185_c1 |
| 7 | c00022_NODE_45_length_2030_cov_70.1602 66 | CO342X | nrps | 1 | 2030 | - | | - |
| 8 | c00026_NODE_20_length_90752_cov_57.841 643 | CO342X | nrps | 1 | 27150 | - | | - |
| 9 | c00028_NODE_14_length_148454_cov_76.38 7250 | CO342X | lantipeptide-nrps | 11199 | 96314 | - | | - |
| 10 | c00032_NODE_7_length_243401_cov_61.083 120 | CO342X | bacteriocin | 63360 | 74310 | - | | - |
| 11 | c00032_NODE_7_length_243401_cov_61.083 120 | CO342X | bacteriocin | 154005 | 164835 | - | | - |
| 12 | c00032_NODE_7_length_243401_cov_61.083 120 | CO342X | nrps | 191175 | 243401 | - | | - |
| 13 | c00034_NODE_17_length_138819_cov_71.08 4877 | CO342X | putative | 44698 | 51855 | - | | - |
| 14 | c00034_NODE_17_length_138819_cov_71.08 4877 | CO342X | t1pks-nrps | 67464 | 138819 | - | | - |
| 15 | c00036_NODE_43_length_3161_cov_133.473 735 | CO342X | nrps | 1 | 3161 | - | | - |
| 16 | c00038_NODE_12_length_162668_cov_63.20 1819 | CO342X | acyl_amino_acids | 62686 | 123579 | - | | - |
| 17 | c00039_NODE_16_length_138882_cov_54.40 0915 | CO342X | t1pks-nrps | 14743 | 64084 | - | | - |
| 18 | c00039_NODE_16_length_138882_cov_54.40 0915 | CO342X | putative | 95443 | 104283 | - | | - |
| 19 | c00040_NODE_2_length_465641_cov_53.519 119 | CO342X | putative | 186168 | 197739 | - | | - |

Table S1. Identified BGC in *Pseudoalteromonas* by antiSMASH (*Continuation*)

| Cluster | Input accession number | Input name *Pseudoalteromonas* sp. | Gene cluster type | from | to | Most similar known cluster (MIBiG) | Similarity | MIBiG BGC-ID |
|---|---|---|---|---|---|---|---|---|
| 20 | c00040_NODE_2_length_465641_cov_53.519119 | CO342X | thiopeptide | 316700 | 354922 | - | | - |
| 21 | c00041_NODE_1_length_550994_cov_55.846605 | CO342X | fatty acid | 792 | 21862 | - | | - |
| 22 | c00041_NODE_1_length_550994_cov_55.846605 | CO342X | putative | 305432 | 310734 | - | | - |
| 23 | c00041_NODE_1_length_550994_cov_55.846605 | CO342X | putative | 477403 | 497639 | - | | - |
| 24 | c00043_NODE_26_length_59499_cov_66.152620 | CO342X | saccharide | 1 | 45690 | O-antigen | 16% | BGC0000784_c1 |
| 25 | c00044_NODE_3_length_376664_cov_66.543338 | CO342X | putative | 35353 | 40268 | - | | - |
| 26 | c00044_NODE_3_length_376664_cov_66.543338 | CO342X | putative | 354091 | 367115 | - | | - |
| 27 | c00046_NODE_8_length_214299_cov_75.176270 | CO342X | nrps | 430 | 55842 | - | | - |
| 28 | c00046_NODE_8_length_214299_cov_75.176270 | CO342X | putative | 77260 | 95787 | - | | - |
| 29 | c00048_NODE_9_length_203384_cov_56.489186 | CO342X | fatty acid | 16889 | 38103 | - | | - |
| 30 | c00048_NODE_9_length_203384_cov_56.489186 | CO342X | fatty acid | 155577 | 176815 | - | | - |
| 31 | c00049_NODE_5_length_307095_cov_59.338703 | CO342X | bacteriocin | 113978 | 124814 | - | | - |
| 32 | c00049_NODE_5_length_307095_cov_59.338703 | CO342X | putative | 192643 | 205171 | - | | - |
| 33 | c00049_NODE_5_length_307095_cov_59.338703 | CO342X | ladderane-nrps | 267716 | 307095 | Alterochromides | 95% | BGC0000299_c1 |
| 34 | c00051_NODE_19_length_119059_cov_73.997319 | CO342X | putative | 20924 | 29279 | Bacillomycin | 20% | BGC0001090_c1 |
| 35 | c00053_NODE_10_length_200666_cov_58.759807 | CO342X | putative | 65829 | 81657 | - | | - |
| 36 | c00054_NODE_18_length_133308_cov_62.288101 | CO342X | nrps | 1 | 27212 | Bromoalterochromides | 14% | BGC0000314_c1 |
| 37 | c00057_NODE_40_length_6534_cov_70.430695 | CO342X | other | 1 | 6534 | - | | - |
| 1 | c00004_NODE_9_length_211179_cov_70.167979 | CO348 | putative | 188544 | 201621 | - | | - |
| 2 | c00005_NODE_14_length_148729_cov_72.514134 | CO348 | putative | 35116 | 40031 | - | | - |
| 3 | c00006_NODE_3_length_380609_cov_76.711850 | CO348 | nrps | 533 | 55945 | - | | - |
| 4 | c00006_NODE_3_length_380609_cov_76.711850 | CO348 | putative | 81457 | 94510 | - | | - |
| 5 | c00006_NODE_3_length_380609_cov_76.711850 | CO348 | lantipeptide-nrps | 239295 | 318184 | - | | |

**Table S1. Identified BGC in *Pseudoalteromonas* by antiSMASH (*Continuation*)**

| Cluster | Input accession number | Input name *Pseudoalteromonas* sp. | Gene cluster type | from | to | Most similar known cluster (MIBiG) | Similarity | MIBiG BGC-ID |
|---|---|---|---|---|---|---|---|---|
| 6 | c00007_NODE_1_length_503294_cov_63.287991 | CO348 | putative | 477 | 9977 | - | | - |
| 7 | c00007_NODE_1_length_503294_cov_63.287991 | CO348 | t1pks-nrps | 174883 | 224224 | - | | - |
| 8 | c00007_NODE_1_length_503294_cov_63.287991 | CO348 | putative | 255943 | 264782 | - | | - |
| 9 | c00007_NODE_1_length_503294_cov_63.287991 | CO348 | fatty acid | 326556 | 347794 | - | | - |
| 10 | c00007_NODE_1_length_503294_cov_63.287991 | CO348 | fatty acid | 465210 | 486424 | - | | - |
| 11 | c00008_NODE_8_length_219423_cov_61.386497 | CO348 | putative | 53017 | 64740 | - | | - |
| 12 | c00012_NODE_6_length_264646_cov_73.560581 | CO348 | saccharide | 41594 | 91570 | Emulsan | 18% | BGC0000760_c1 |
| 13 | c00012_NODE_6_length_264646_cov_73.560581 | CO348 | fatty acid-t1pks-nrps | 160825 | 264646 | - | | - |
| 14 | c00016_NODE_10_length_208930_cov_69.532763 | CO348 | putative | 71189 | 90845 | - | | - |
| 15 | c00018_NODE_4_length_314892_cov_66.600618 | CO348 | bacteriocin | 121569 | 132405 | - | | - |
| 16 | c00018_NODE_4_length_314892_cov_66.600618 | CO348 | putative | 200538 | 213066 | - | | - |
| 17 | c00018_NODE_4_length_314892_cov_66.600618 | CO348 | ladderane-nrps | 275404 | 314892 | Alterochromides | 95% | BGC0000299_c1 |
| 18 | c00019_NODE_45_length_1795_cov_171.722870 | CO348 | other | 1 | 1795 | - | | - |
| 19 | c00027_NODE_18_length_122393_cov_75.665127 | CO348 | t1pks-nrps | 605 | 85780 | Enterobactin | 8% | BGC0000343_c1 |
| 20 | c00029_NODE_7_length_262814_cov_66.321357 | CO348 | putative | 18754 | 24056 | - | | - |
| 21 | c00029_NODE_7_length_262814_cov_66.321357 | CO348 | putative | 192593 | 214183 | - | | - |
| 22 | c00035_NODE_15_length_133296_cov_67.349675 | CO348 | nrps | 1 | 26760 | Bromoalterochromides | 14% | BGC0000314_c1 |
| 23 | c00036_NODE_19_length_112961_cov_63.342390 | CO348 | fatty acid | 772 | 21842 | - | | - |
| 24 | c00038_NODE_40_length_7178_cov_89.174722 | CO348 | nrps | 1 | 7178 | - | | - |
| 25 | c00043_NODE_13_length_149429_cov_69.951951 | CO348 | putative | 92383 | 99345 | - | | - |
| 26 | c00043_NODE_13_length_149429_cov_69.951951 | CO348 | putative | 111972 | 121333 | - | | - |
| 27 | c00046_NODE_2_length_401822_cov_70.165380 | CO348 | bacteriocin | 250977 | 261927 | - | | - |
| 28 | c00046_NODE_2_length_401822_cov_70.165380 | CO348 | bacteriocin | 338107 | 348937 | - | | - |

**Table S1. Identified BGC in *Pseudoalteromonas* by antiSMASH (*Continuation*)**

| Cluster | Input accession number | Input name *Pseudoalteromonas* sp. | Gene cluster type | from | to | Most similar known cluster (MIBiG) | Similarity | MIBiG BGC-ID |
|---|---|---|---|---|---|---|---|---|
| 29 | c00046_NODE_2_length_401822_cov_70.165380 | CO348 | nrps | 373078 | 401822 | - | | - |
| 30 | c00048_NODE_23_length_78873_cov_67.657046 | CO348 | other | 1 | 22050 | - | | - |

**Table S2. MultiGeneBlast Results per category**

| Query Cluster | Query antiSMASH category | Hit Cluster | Hit antiSMAH Category | Total Score | Cumulative Blast score | BGC similarity (%) * |
|---|---|---|---|---|---|---|
| CO342X_Cluster002 | NRPS | CO342X_Cluster002 | NRPS | 4.5 | 3991 | 100 |
| | | CO348_cluster29 | NRPS | 3 | 750 | 31 |
| | | CO348_cluster005 | lantipeptide-nrps | 2 | 1634 | 40 |
| | | CO342X_cluster009 | lantipeptide-nrps | 2 | 1633 | 40 |
| | | CO342X_cluster014 | T1pks-nrps | 2 | 1460 | 40 |
| | | CO348_cluster013 | Fatty acid-T1pks-nrps | 2 | 1442 | 40 |
| | | CO342X_cluster012 | NRPS | 2 | 1341 | 35 |
| | | CO342X_cluster027 | NRPS | 2 | 1118 | 53 |
| | | CO348_cluster003 | NRPS | 2 | 1107 | 46 |
| | | CO348_cluster019 | T1pks-nrps | 2 | 438 | 45 |
| | | CO342X_cluster006 | NRPS | 2 | 201 | 47 |
| | | CO348_cluster24 | NRPS | 1 | 678 | 39 |
| | | CO342X_cluster006 | NRPS | 1 | 50 | 32 |
| CO342X_Cluster005 | NRPS | CO348_cluster005 | Lantipeptide-nrps | 1 | 1490 | 41 |
| | | CO342X_cluster009 | Lantipeptide-nrps | 1 | 1484 | 40 |
| | | CO342X_cluster014 | T1pks-nrps | 1 | 1331 | 34 |
| | | CO348_cluster013 | Fatty acid-T1pks-nrps | 1 | 1325 | 34 |
| | | CO342X_cluster012 | NRPS | 1 | 1203 | 31 |
| | | CO342X_cluster002 | NRPS | 1 | 1170 | 52 |
| | | CO348_cluster24 | NRPS | 1 | 674 | 39 |
| | | CO348_cluster019 | T1pks-nrps | 1 | 409 | 31 |
| | | CO348_cluster019 | T1pks-nrps | 1 | 64 | 36 |
| CO342X_Cluster006 | NRPS | CO342X_cluster006 | NRPS | 54 | 29779 | 100 |
| | | CO348_cluster019 | T1pks-nrps | 25.5 | 10344 | 46 |
| | | CO342X_cluster014 | T1pks-nrps | 2 | 330 | 37 |
| | | CO348_cluster013 | Fatty acid-T1pks-nrps | 2 | 325 | 37 |
| | | CO348_cluster24 | NRPS | 2 | 259 | 31 |
| | | CO342X_cluster002 | NRPS | 2 | 250 | 32 |
| | | CO342X_cluster027 | NRPS | 1 | 168 | 37 |
| | | CO348_cluster003 | NRPS | 1 | 165 | 44 |
| | | CO348_cluster29 | NRPS | 1 | 123 | 34 |
| | | CO348_cluster013 | Fatty acid-T1pks-nrps | 1 | 108 | 33 |

* The BGC similarity percentage is shown for the core biosynthetic gene.

**Table S2. MultiGeneBlast Results per category** (*Continuation*)

| Query Cluster | Query antiSMASH category | Hit Cluster | Hit antiSMAH Category | Total Score | Cumulative Blast score | BGC similarity (%) * |
|---|---|---|---|---|---|---|
| CO342X_Cluster007 | NRPS | CO348_cluster019 | T1pks-nrps | 1 | 150 | 45 |
| CO342X_Cluster008 | NRPS | CO342X_Cluster008 | NRPS | 25.5 | 17615 | 100 |
| | | CO348_cluster019 | T1pks-nrps | 4 | 700 | 43 |
| CO342X_Cluster009 | Lantipeptide-nrps | CO342X_Cluster009 | Lantipeptide-nrps | 82 | 52530 | 100 |
| | | CO348_cluster005 | Lantipeptide-nrps | 69 | 49011 | 99 |
| | | CO348_cluster019 | T1pks-nrps | 4 | 582 | 33 |
| | | CO342X_cluster014 | Lantipeptide-nrps | 3 | 2213 | 32 |
| | | CO348_cluster013 | Fatty acid-T1pks-nrps | 3 | 2212 | 32 |
| | | CO342X_cluster002 | NRPS | 3 | 1612 | 34 |
| | | CO348_cluster29 | NRPS | 3 | 259 | 0 |
| | | CO342X_cluster012 | NRPS | 2 | 1318 | 31 |
| | | CO348_cluster24 | NRPS | 2 | 1214 | 32 |
| | | CO342X_cluster008 | NRPS | 1 | 443 | 0 |
| CO342X_Cluster012 | NRPS | CO342X_cluster012 | NRPS | 40.5 | 28518 | 100 |
| | | CO348_cluster29 | NRPS | 12 | 3883 | 99 |
| | | CO342X_cluster002 | NRPS | 3 | 1338 | 34 |
| | | CO342X_cluster009 | Lantipeptide-nrps | 3 | 1317 | 31 |
| | | CO348_cluster005 | Lantipeptide-nrps | 3 | 1316 | 31 |
| | | CO348_cluster019 | T1pks-nrps | 3 | 157 | 33 |
| | | CO348_cluster24 | NRPS | 2 | 481 | 34 |
| | | CO348_cluster013 | Fatty acid-T1pks-nrps | 2 | 310 | 0 |
| | | CO348_cluster019 | T1pks-nrps | 1 | 268 | 0 |
| CO342X_Cluster014 | T1pks-nrps | CO342X_cluster014 | T1pks-nrps | 59 | 46127 | 100 |
| | | CO348_cluster013 | Fatty acid-T1pks-nrps | 55 | 43681 | 97 |
| | | CO348_cluster019 | T1pks-nrps | 4.5 | 1507 | 40 |
| | | CO342X_cluster009 | Lantipeptide-nrps | 3 | 2244 | 32 |
| | | CO348_cluster005 | Lantipeptide-nrps | 3 | 2241 | 31 |
| | | CO342X_cluster002 | NRPS | 3 | 1461 | 35 |
| | | CO348_cluster24 | NRPS | 2 | 872 | 32 |
| | | CO342X_cluster008 | NRPS | 1 | 516 | 0 |
| | | CO342X_cluster006 | NRPS | 1 | 71 | 32 |

* The BGC similarity percentage is shown for the core biosynthetic gene.

**Table S2. MultiGeneBlast Results per category** (*Continuation*)

| Query Cluster | Query antiSMASH category | Hit Cluster | Hit antiSMAH Category | Total Score | Cumulative Blast score | BGC similarity (%) * |
|---|---|---|---|---|---|---|
| CO342X_Cluster015 | NRPS | CO348_cluster005 | Lantipeptide-nrps | 1 | 1311 | 38 |
| | | CO342X_cluster009 | Lantipeptide-nrps | 1 | 1309 | 38 |
| | | CO342X_cluster014 | T1pks-nrps | 1 | 1277 | 39 |
| | | CO348_cluster013 | Fatty acid-T1pks-nrps | 1 | 1268 | 39 |
| | | CO348_cluster019 | T1pks-nrps | 1 | 1244 | 31 |
| | | CO342X_cluster002 | NRPS | 1 | 1185 | 55 |
| | | CO342X_cluster012 | NRPS | 1 | 1111 | 32 |
| | | CO342X_cluster027 | NRPS | 1 | 972 | 35 |
| | | CO348_cluster003 | NRPS | 1 | 969 | 35 |
| | | CO348_cluster24 | NRPS | 1 | 585 | 31 |
| CO342X_Cluster017 | T1pks-nrps | CO342X_Cluster017 | T1pks-nrps | 56 | 31418 | 100 |
| | | CO348_cluster007 | T1pks-nrps | 37.5 | 24594 | 98 |
| CO342X_Cluster027 | NRPS | CO342X_cluster027 | NRPS | 68.5 | 35514 | 100 |
| | | CO348_cluster003 | NRPS | 65.5 | 34569 | 99 |
| | | CO348_cluster017 | ladderane-nrps | 4 | 1057 | 34 |
| | | CO342X_cluster002 | NRPS | 3 | 1112 | 31 |
| | | CO342X_cluster033 | ladderane-nrps | 3 | 752 | 34 |
| CO342X_Cluster033 | ladderane-nrps | CO342X_cluster033 | ladderane-nrps | 31 | 17938 | 100 |
| | | CO348_cluster017 | ladderane-nrps | 29.5 | 17701 | 99 |
| | | CO348_cluster003 | NRPS | 2 | 752 | 34 |
| | | CO342X_cluster027 | NRPS | 2 | 752 | 34 |
| | | CO348_cluster013 | Fatty acid-T1pks-nrps | 2 | 256 | 32 |
| | | CO348_cluster013 | Fatty acid-T1pks-nrps | 1 | 434 | 32 |
| CO342X_Cluster036 | NRPS | CO342X_cluster036 | NRPS | 25 | 16740 | 100 |
| | | CO348_cluster22 | NRPS | 16 | 11798 | 98 |
| | | CO342X_cluster027 | NRPS | 8.5 | 4403 | 0 |
| | | CO348_cluster019 | T1pks-nrps | 1 | 286 | 0 |
| | | CO348_cluster019 | T1pks-nrps | 1 | 58 | 37 |
| CO348_Cluster003 | NRPS | CO348_cluster003 | NRPS | 66.5 | 35149 | 100 |
| | | CO342X_cluster027 | NRPS | 64 | 345117 | 99 |
| | | CO348_cluster017 | ladderane-nrps | 4 | 1054 | 34 |
| | | CO342X_cluster002 | NRPS | 3 | 1099 | 31 |
| | | CO342X_cluster033 | ladderane-nrps | 3 | 753 | 34 |

* The BGC similarity percentage is shown for the core biosynthetic gene.

**Table S2. MultiGeneBlast Results per category** (*Continuation*)

| Query Cluster | Query antiSMASH category | Hit Cluster | Hit antiSMAH Category | Total Score | Cumulative Blast score | BGC similarity (%) * |
|---|---|---|---|---|---|---|
| CO348_Cluster005 | Lantipeptide-nrps | CO348_cluster005 | Lantipeptide-nrps | 71.5 | 50088 | 100 |
| | | CO342X_cluster009 | Lantipeptide-nrps | 69 | 49060 | 99 |
| | | CO348_cluster019 | T1pks-nrps | 4 | 1010 | 31 |
| | | CO342X_cluster014 | T1pks-nrps | 3 | 2209 | 32 |
| | | CO348_cluster013 | fatty_acid-T1pks-nrps | 3 | 2207 | 32 |
| | | CO342X_cluster002 | NRPS | 3 | 1617 | 35 |
| | | CO342X_cluster012 | NRPS | 3 | 1316 | 31 |
| | | CO348_cluster24 | NRPS | 2 | 1211 | 36 |
| | | CO348_cluster29 | NRPS | 2 | 117 | 32 |
| | | CO342X_cluster008 | NRPS | 1 | 443 | 36 |
| CO348_Cluster007 | T1pks-nrps | CO348_Cluster007 | T1pks-nrps | 62 | 30669 | 100 |
| | | CO342X_cluster017 | T1pks-nrps | 37.5 | 24594 | 98 |
| CO348_Cluster013 | Fatty acid-T1pks-nrps | CO348_cluster013 | Fatty acid-T1pks-nrps | 109.5 | 64829 | 100 |
| | | CO342X_cluster014 | T1pks-nrps | 55 | 43696 | 95 |
| | | CO348_cluster019 | T1pks-nrps | 4 | 1220 | 34 |
| | | CO342X_cluster009 | Lantipeptide-nrps | 3 | 2241 | 31 |
| | | CO348_cluster005 | Lantipeptide-nrps | 3 | 2238 | 31 |
| | | CO342X_cluster002 | NRPS | 3 | 1444 | 35 |
| | | CO348_cluster024 | NRPS | 2 | 869 | 33 |
| | | CO348_cluster017 | ladderane-nrps | 2 | 563 | 34 |
| | | CO342X_cluster033 | ladderane-nrps | 2 | 562 | 34 |
| | | CO342X_cluster006 | NRPS | 2 | 362 | 31 |
| | | CO342X_cluster012 | NRPS | 2 | 310 | 0 |
| | | CO342X_cluster008 | NRPS | 1 | 515 | 0 |
| | | CO342X_cluster006 | NRPS | 1 | 70 | 37 |
| CO348_Cluster017 | ladderane-nrps | CO348_cluster017 | ladderane-nrps | 29.5 | 17854 | 100 |
| | | CO342X_cluster033 | ladderane-nrps | 29.5 | 17701 | 99 |
| | | CO348_cluster003 | NRPS | 2 | 755 | 34 |
| | | CO342X_cluster027 | NRPS | 2 | 755 | 34 |
| | | CO348_cluster005 | Lantipeptide-nrps | 2 | 382 | 34 |
| | | CO348_cluster013 | fatty_acid-T1pks-nrps | 2 | 259 | 32 |
| | | CO348_cluster013 | fatty_acid-T1pks-nrps | 1 | 436 | 32 |

* The BGC similarity percentage is shown for the core biosynthetic gene.

**Table S2. MultiGeneBlast Results per category** (*Continuation*)

| Query Cluster | Query antiSMASH category | Hit Cluster | Hit antiSMAH Category | Total Score | Cumulative Blast score | BGC similarity (%) * |
|---|---|---|---|---|---|---|
| CO348_Cluster019 | T1pks-nrps | CO348_cluster019 | T1pks-nrps | 73 | 58406 | 100 |
| | | CO342X_cluster006 | NRPS | 39.5 | 17351 | 42 |
| | | CO342X_cluster002 | NRPS | 6 | 438 | 36 |
| | | CO342X_cluster014 | T1pks-nrps | 4.5 | 1508 | 37 |
| | | CO348_cluster005 | Lantipeptide-nrps | 4 | 1013 | 34 |
| | | CO348_cluster013 | fatty_acid-T1pks-nrps | 3 | 1219 | 36 |
| | | CO342X_cluster008 | NRPS | 3 | 706 | 31 |
| | | CO342X_cluster009 | Lantipeptide-nrps | 3 | 583 | 34 |
| | | CO348_cluster29 | NRPS | 2.5 | 434 | 33 |
| | | CO342X_cluster012 | NRPS | 2 | 425 | 33 |
| | | CO348_cluster003 | NRPS | 2 | 119 | 32 |
| | | CO342X_cluster027 | NRPS | 1 | 119 | 32 |
| CO348_Cluster022 | NRPS | CO342X_cluster036 | NRPS | 25 | 16200 | 98 |
| | | CO348_cluster22 | NRPS | 16 | 12030 | 100 |
| | | CO342X_cluster027 | NRPS | 8.5 | 4511 | 0 |
| | | CO348_cluster019 | T1pks-nrps | 1 | 58 | 36 |
| CO348_Cluster024 | NRPS | CO348_cluster24 | NRPS | 2.5 | 3746 | 100 |
| | | CO342X_cluster014 | T1pks-nrps | 2.5 | 1116 | 33 |
| | | CO342X_cluster009 | Lantipeptide-nrps | 2 | 1215 | 36 |
| | | CO348_cluster005 | Lantipeptide-nrps | 2 | 1211 | 33 |
| | | CO348_cluster013 | fatty_acid-T1pks-nrps | 2 | 863 | 33 |
| | | CO348_cluster019 | T1pks-nrps | 2 | 507 | 33 |
| | | CO342X_cluster012 | NRPS | 2 | 481 | 34 |
| | | CO342X_cluster006 | NRPS | 1 | 46 | 31 |
| CO348_Cluster029 | NRPS | CO348_cluster029 | NRPS | 40 | 16127 | 100 |
| | | CO342X_cluster012 | NRPS | 12 | 3883 | 99 |
| | | CO348_cluster019 | T1pks-nrps | 2.5 | 434 | 33 |
| | | CO342X_cluster002 | NRPS | 2 | 740 | 31 |
| | | CO348_cluster005 | Lantipeptide-nrps | 1 | 80 | 0 |
| | | CO342X_cluster009 | Lantipeptide-nrps | 1 | 44 | 0 |

* The BGC similarity percentage is shown for the core biosynthetic gene.

**Table S2. MultiGeneBlast Results per category** (*Continuation*)

| Query Cluster | Query antiSMASH category | Hit Cluster | Hit antiSMAH Category | Total Score | Cumulative Blast score | BGC similarity (%) * |
|---|---|---|---|---|---|---|
| CO109Y_cluster005 | Bacteriocin | CO109Y_cluster005 | Bacteriocin | 16 | 6168 | 100 |
| | | CO348_cluster015 | Bacteriocin | 16 | 3394 | 72 |
| | | CO342X_cluster031 | Bacteriocin | 15.5 | 3411 | 72 |
| | | CO325X_cluster014 | Bacteriocin | 11.5 | 2108 | 72 |
| | | CO302Y_cluster008 | Bacteriocin | 8.5 | 3025 | 83 |
| | | CO302Y_cluster008 | Bacteriocin | 8.5 | 3025 | 83 |
| CO133X_cluster008 | Bacteriocin | CO133X_cluster008 | Bacteriocin | 10 | 3569 | 100 |
| | | CO302Y_cluster008 | Bacteriocin | 10 | 3567 | 100 |
| | | CO109Y_cluster005 | Bacteriocin | 8.5 | 3026 | 83 |
| | | CO348_cluster015 | Bacteriocin | 8.5 | 2023 | 69 |
| | | CO342X_cluster031 | Bacteriocin | 8 | 2043 | 70 |
| | | CO325X_cluster014 | Bacteriocin | 4 | 738 | 68 |
| CO302Y_cluster008 | Bacteriocin | CO302Y_cluster008 | Bacteriocin | 10 | 3569 | 100 |
| | | CO133X_cluster008 | Bacteriocin | 10 | 3567 | 100 |
| | | CO109Y_cluster005 | Bacteriocin | 8.5 | 3026 | 83 |
| | | CO348_cluster015 | Bacteriocin | 8.5 | 2023 | 69 |
| | | CO342X_cluster031 | Bacteriocin | 8 | 2043 | 70 |
| | | CO325X_cluster014 | Bacteriocin | 4 | 738 | 68 |
| CO325X_cluster014 | Bacteriocin | CO325X_cluster014 | Bacteriocin | 16 | 5199 | 100 |
| | | CO109Y_cluster005 | Bacteriocin | 11.5 | 2193 | 72 |
| | | CO342X_cluster031 | Bacteriocin | 9.5 | 1802 | 70 |
| | | CO348_cluster015 | Bacteriocin | 9.5 | 1796 | 69 |
| | | CO302Y_cluster008 | Bacteriocin | 4 | 738 | 68 |
| | | CO133X_cluster008 | Bacteriocin | 4 | 738 | 68 |
| CO342X_cluster010 | Bacteriocin | CO342X_cluster010 | Bacteriocin | 8.5 | 5220 | 100 |
| | | CO348_cluster27 | Bacteriocin | 8.5 | 4831 | 97 |
| CO342X_cluster011 | Bacteriocin | CO342X_cluster011 | Bacteriocin | 13 | 4748 | 100 |
| | | CO348_cluster28 | Bacteriocin | 10.5 | 4450 | 98 |

* The BGC similarity percentage is shown for the core biosynthetic gene.

**Table S2. MultiGeneBlast Results per category** (*Continuation*)

| Query Cluster | Query antiSMASH category | Hit Cluster | Hit antiSMAH Category | Total Score | Cumulative Blast score | BGC similarity (%) * |
|---|---|---|---|---|---|---|
| CO342X_cluster031 | Bacteriocin | CO342X_cluster031 | Bacteriocin | 17.5 | 6219 | 100 |
| | | CO348_cluster015 | Bacteriocin | 15.5 | 5922 | 98 |
| | | CO109Y_cluster005 | Bacteriocin | 15.5 | 3556 | 72 |
| | | CO325X_cluster014 | Bacteriocin | 9.5 | 1818 | 70 |
| | | CO302Y_cluster008 | Bacteriocin | 8 | 2043 | 70 |
| | | CO133X_cluster008 | Bacteriocin | 8 | 2043 | 70 |
| CO348_cluster015 | Bacteriocin | CO348_cluster015 | Bacteriocin | 16 | 6090 | 100 |
| | | CO109Y_cluster005 | Bacteriocin | 16 | 3547 | 72 |
| | | CO342X_cluster031 | Bacteriocin | 15.5 | 5938 | 98 |
| | | CO325X_cluster014 | Bacteriocin | 9.5 | 1810 | 69 |
| | | CO302Y_cluster008 | Bacteriocin | 8.5 | 2028 | 69 |
| | | CO133X_cluster008 | Bacteriocin | 8.5 | 2028 | 69 |
| CO348_cluster027 | Bacteriocin | CO348_cluster027 | Bacteriocin | 8.5 | 5229 | 100 |
| | | CO342X_cluster010 | Bacteriocin | 8.5 | 4831 | 97 |
| CO348_cluster028 | Bacteriocin | CO348_cluster028 | Bacteriocin | 14.5 | 4931 | 100 |
| | | CO342X_cluster011 | Bacteriocin | 10.5 | 4450 | 98 |

* The BGC similarity percentage is shown for the core biosynthetic gene.

**Table S2. MultiGeneBlast Results per category** (*Continuation*)

| Query Cluster | Query antiSMASH category | Hit Cluster | Hit antiSMAH Category | Total Score | Cumulative Blast score | BGC similarity (%) * |
|---|---|---|---|---|---|---|
| CO109Y_cluster012 | Siderophore | CO109Y_cluster012 | Siderophore | 16 | 7274 | 100 |
| | | CO325X_cluster002 | Siderophore | 5 | 2506 | 73 |
| | | CO302Y_cluster012 | Siderophore | 3.5 | 1417 | 42 |
| | | CO133X_cluster016 | Siderophore | 3.5 | 1417 | 42 |
| CO133X_cluster016 | Siderophore | CO302Y_cluster012 | Siderophore | 13 | 7098 | 100 |
| | | CO133X_cluster016 | Siderophore | 13 | 7098 | 100 |
| | | CO109Y_cluster012 | Siderophore | 3.5 | 1443 | 42 |
| | | CO325X_cluster002 | Siderophore | 3 | 1085 | 41 |
| CO302Y_cluster012 | Siderophore | CO302Y_cluster012 | Siderophore | 13 | 7098 | 100 |
| | | CO133X_cluster016 | Siderophore | 13 | 7098 | 100 |
| | | CO109Y_cluster012 | Siderophore | 3.5 | 1443 | 42 |
| | | CO325X_cluster002 | Siderophore | 3 | 1085 | 41 |
| CO325X_cluster002 | Siderophore | CO325X_cluster002 | Siderophore | 8.5 | 6544 | 100 |
| | | CO109Y_cluster012 | Siderophore | 5 | 22522 | 73 |
| | | CO302Y_cluster012 | Siderophore | 2 | 1063 | 41 |
| | | CO133X_cluster016 | Siderophore | 2 | 1063 | 41 |

* The BGC similarity percentage is shown for the core biosynthetic gene.

**Table S3. Summary of BGC found in *Pseudoalteromonas* species according to antiSMASH database**

| Categories | Number of BCG | Found in | Most similar MIBiG clusters |
|---|---|---|---|
| Aryl polyene | 45 | *P. agarivorans, P. aliena ,P. arabiensis, P. gelatinilytica, P. haloplanktis, P. lipolytica, P. luteoviolaceae, P. nigrifaciens,P. piratica, P. prydzensis, P. ruthenica, P. shioyasakiensis, P. spongiae, P.translucida Pseudoalteromonas* sp. (unclassified) | APEVf |
| Bacteriocin or other unspecified RiPP | 186 | *P. agarivorans, P. aliena, P. amylolytica, P. arabiensis, P. arctica, P. atlantica, P. byunsanensis, P. carrageenovora, P. citrea, P. denitrificans, P. elyakovii, P. espejiana, P. flavipulchra, P. fuliginea, P. gelatinilytica, P. haloplanktis, P. issachenkonii, P. lipolytica, P. luteaoviolaceae, P. marina,P. neustonica, P. nigrifaciens , P. phenolica P. piratica, P. piscicida, P. porphyrae,P. prydzensis, P. rubra, P. ruthenica, P. shioyasakiensis, P. spongiae, P. teatraodonis, P. translucida, P. tunicata, P. undina, Pseudoalteromonas* sp. (unclassified). | |
| Homoserine lactone | 12 | *P. atlantica, P. luteoviolaceae, P. rubra, Pseudoalteromonas* sp. (unclassified) | |
| Hybrid cluster: Aryl polyene & Lanthipeptide | 1 | *P. denitrificans* | APEVf |
| Hybrid cluster: Aryl polyene & Nonribosomal peptide | 9 | *P. flavipulchra, P. luteoviolaceae, P. piscicida Pseudoalteromonas* sp. (unclassified) | APEVf, Alterochromides, Bromoalterochromides |
| Hybrid cluster: Aryl polyene & Resorcinol | 9 | *P. arctica, P. neustonica,P. porphyrae, P. spongiae, Pseudoalteromonas* sp. (unclassified) | APEVf, Flexirubin |
| Hybrid cluster: Bacteriocin or other unspecified RiPP & Nonribosomal peptide | 1 | *P. aliena* | |
| Hybrid cluster: Bacteriocin or other unspecified RiPP & Nonribosomal peptide & Type I polyketide | 1 | *P. rubra* | |
| Hybrid cluster: Butyrolactone & Homoserine lactone & Nonribosomal peptide | 1 | *Pseudoalteromonas sp.* (unclassified) | |
| Hybrid cluster: Butyrolactone & Type I polyketide | 1 | *P. byunsanensis* | |
| Hybrid cluster: hglE-type polyketide & Nonribosomal peptide & Trans-AT polyketide | 2 | *P. luteoviolaceae* | Thiomarinol, Calyculin |
| Hybrid cluster: hglE-type polyketide & Polyunsaturated fatty acid | 1 | *P. denitrificans* | Eicosapentaenoicacid-like compound |
| Hybrid cluster: hglE-type polyketide & Polyunsaturated fatty acid & Type I polyketide | 1 | *Pseudoalteromonas sp.* (unclassified) | Eicosapentaenoicacid-like compound |
| Hybrid cluster: Homoserine lactone & N-acyl amino acid cluster | 1 | *P. luteoviolaceae* | |

**Table S3. Summary of BGC found in *Pseudoalteromonas* species according to antiSMASH database** *(Continuation)*

| **Categories** | **Number of BCG** | **Found in** | **Most similar MIBiG clusters** |
|---|---|---|---|
| Hybrid cluster: Homoserine lactone & Type I polyketide | 2 | *P. citrea, Pseudoalteromonas* sp. (unclassified) | |
| Hybrid cluster: Indole & Type III polyketide | 5 | *P. luteoviolaceae* | Violacein |
| Hybrid cluster: Ladderane & Lanthipeptide | 1 | *P. luteoviolaceae* | |
| Hybrid cluster: Ladderane & Nonribosomal peptide | 7 | *P. elyakovii, P. piscicida, P. luteoviolaceae Pseudoalteromonas* sp. (unclassified) | Altherochromides, Bromoalterochromides |
| Hybrid cluster: Lanthipeptide & Nonribosomal peptide | 6 | *P. elyakovii, P. piscicida, P. rubra, Pseudoalteromonas* sp. (unclassified) | |
| Hybrid cluster: Lanthipeptide & Nonribosomal peptide & Type I polyketide | 2 | *P. piscicida* | Colibactin |
| Hybrid cluster: Lanthipeptide & Trans-AT polyketide | 1 | *P. luteoviolaceae* | Cystobactamide |
| Hybrid cluster: Nonribosomal peptide & Thiopeptide | 1 | *P. rubra* | |
| Hybrid cluster: Nonribosomal peptide & Trans-AT polyketide | 1 | *P. rubra* | |
| Hybrid cluster: Nonribosomal peptide & Type I polyketide | 70 | *P. amylolytica, P. byunsanensis, P. citrea, P. denitrificans, P. elyakovii, P. flavipulchra, P. luteoviolaceae, P. piscicida, P. prydzensis, P. rubra, Pseudoalteromonas* sp. (unclassified) | Turnerbactin,Streptomycin, Serobactins, Hectochlorin, Cystobactamide, Griseobactin,Enterobactin, Pyoverdine, |
| Hybrid cluster: Nonribosomal peptide & Type III polyketide | 2 | *P. luteoviolaceae, P. piscicida* | Feglymycin |
| Indole | 10 | *P. amylolytica, P. byunsanensis, P. luteoviolaceae, P. tunicata, Pseudoalteromonas* sp. (unclassified) | Violacein |
| Lanthipeptide | 23 | *P. amylolytica, P. byunsanensis, P. citrea, P. espejiana, P. flavipulchra, P. haloplanktis, P. luteoviolaceae, P. phenolica, P. piscicida, P. rubra, Pseudoalteromonas* sp. (unclassified) | Xenocyloins |
| N-acyl amino acid cluster | 4 | *P. elyakovii, P. piscicida, P. luteoviolaceae, Pseudoalteromonas* sp. (unclassified) | |

**Table S3. Summary of BGC found in *Pseudoalteromonas* species according to antiSMASH database** *(Continuation)*

| **Categories** | **Number of BCG** | **Found in** | **Most similar MIBiG clusters** |
|---|---|---|---|
| Nonribosomal Peptide | 178 | *P. amylolytica, P. byunsanensis, P. citrea, P. denitrificans, P. elyakovii, P. flavipulchra,P. fuliginea, P. luteoviolaceae,P. neustonica, P. phenolica, P. piscicida, P. porphyrae, P. rubra, P. tunicata Pseudoalteromonas* sp. (unclassified) | Taxlllaid, Syringomycin, Bacillibactin, Bromoalterochromides, Orfamide, Colibactin, Cupriachelin, Alterochromides, Cahuitamycins, Dorrigocin/migrastatin,Stenothricin, Kalimantacin/batumin, Turnerbactin, Vibrobactin, Bongkrekicacid, Pyoverdine, Vulnibactin, Enterobactin, Griseobactin, Bacitracin,Heterobactin, Nunapeptin/nunamycin, Griselimycin, Syringomycin, Mycobactin, WAP-8294A2(lotilibcin), Syringopeptin |
| Other | 35 | *P. amylolytica, P. byunsanensis, P. citrea, P. fuliginea, P. luteoviolaceae ,P. nigrifaciens, P. piscicida, P. rubra, P. tunicata, Pseudoalteromonas* sp. (unclassified) | Pyoverdine, Auricindeoxysugarmoieties, Indigoidine, Prodigiosin |
| Phosphonate | 1 | *P. byunsanensis* | |
| Polybrominated diphenyl ether | 1 | *P. phenolica* | Bromophenols/bromopyrroles |
| Resorcinol | 3 | *P. ruthenica* | |
| Siderophore | 50 | *P. agarivorans, P. aliena ,P. amylolytica, P. arabiensis,P. byunsanensis, P. carrageenovora, P. espejiana, P. gelatinilytica, P. haloplanktis, P. issachenkonii, P. lipolytica,P. piratica, P. prydzensis, P. ruthenica, P. shioyasakiensis, P. spongiae, P. tetraodonis ,P. tunicata, Pseudoalteromonas* sp. (unclassified) | DesferrioxamineB |
| Thiopeptide | 7 | *P. citrea,P. elyakovii, P. flavipulchra, P. luteaoviolaceae, Pseudoalteromonas* sp. (unclassified) | |
| Type I polyketide | 3 | *P. citrea, P. luteaoviolaceae, P. rubra* | |
| Type II polyketide | 4 | *P. luteoviolaceae* | |
| Type III polyketide | 10 | *P. citrea, P. luteaoviolaceae, P. rubra, Pseudoalteromonas* sp. (unclassified) | |

**Table S4. MS1 peaks considered for MS2 fragmentation data.**

| Time | Collision RF | Transfer Time | Collision |
|---|---|---|---|
| 0 | 450.0 | 70.0 | 125 |
| 25 | 550.0 | 75.0 | 100 |
| 50 | 800.0 | 90.0 | 100 |
| 75 | 1100.0 | 95.0 | 75 |

**Table S5. Collision-induced dissociation (CID) energies for MS/MS data acquisition**

| Type | Mass | Width | Collision | Charge State |
|---|---|---|---|---|
| Base | 100.00 | 4.00 | 22.00 | 1 |
| Base | 100.00 | 4.00 | 18.00 | 2 |
| Base | 300.00 | 5.00 | 27.00 | 1 |
| Base | 300.00 | 5.00 | 22.00 | 2 |
| Base | 500.00 | 6.00 | 35.00 | 1 |
| Base | 500.00 | 6.00 | 30.00 | 2 |
| Base | 1000.00 | 8.00 | 45.00 | 1 |
| Base | 1000.00 | 8.00 | 35.00 | 2 |
| Base | 2000.00 | 10.00 | 50.00 | 1 |
| Base | 2000.00 | 10.00 | 50.00 | 2 |

## Table S6. Observed CID fragment ions for characterized bromoalterochromides

| Open species | Fragment* | Bromoalterochromide A/A' m/z 844.2900 $[M + H]^+$ (1,2) | Dibromoalterochromide A/A' m/z 922.1996 $[M + H]^+$ (3,4) | Bromoalterochromide B/B' m/z 870.3061$[M + H]^+$ (5,6) | Dibromoalterochromide B/B' m/z 948.2175 $[M + H]^+$ (7,8) | Bromoalterochromide D/D' m/z 858.3049 $[M + H]^+$ (9,10) | Dibromoalterochromide D/D' m/z 936.2157 $[M + H]^+$ (11,12) |
|---|---|---|---|---|---|---|---|
| **Dehydration** | M-OH | 827/829 | 905/907/909 | 853/855 | 931/933/935 | 841/843 | 919/921/923 |
| **Isomeric open specie A** | $a_1$ | - | 435/437/439 | - | - | 358/360 (low) | - |
| | $a_2$ | 457/459 | 535/537/539 | 483/485 | 561/563/565 | 471/473 | 549/551/553 |
| | $a_4$ | 685/687 | 763/765/767 (low) | - | - | 699/701 | 777/779/781 (low) |
| | $a_5$ | 798/800 | - | - | - | - | - |
| | $b_0$ | 303/305 | 381/383/385 | 329/331 | 406/408/410 | 303/305 | 380/382/384 |
| | $b_1$ | 386/388 | 464/466/468 | 412/414 | 489/491/493 | 386/388 | 464/466/468 |
| | $b_2$ | 485/487 | 563/565/567 | 511/513 | 589/591/593 (low) | 499/501 | 577/579/581 |
| | $b_3$ | 599/601 | 677/679/681 | 625/627 | 703/705/707 (low) | 613/615 | 691/693/695 |
| | $b_4$ | 713/715 | 791/793/795 | - | - | 727/729 | 805/807/809 |
| | $y_1$ | 131 | 131 | 131 | 131 | 131 | 131 |
| | $y_2$ | 246 | 246 | 246 | 246 | 246 | 246 |
| | $y_3$ | 360 | 360 | 360 | 360 | 360 | 360 |
| | $y_4$ | 459 | 459 | 459 | 459 | 473 | 473 |
| | $y_5$ | 542 | 542 | 542 | 542 | 556 | 556 |
| | $c_1$ | 404/406 | 482/484/486 | - | - | 404/406 | 482/484/486 |
| | $c_3$ | 617/619 | - | - | - | 631/633 (low) | - |
| | $c_4$ | 731/733 | - | | | - | - |
| | $z_2$ | 229 | 229 | 229 | 229 | 229 | 229 |
| | $z_3$ | 343 | 343 | 343 | 343 | 343 | 343 |
| | $z_4$ | 442 | 442 | 442 | 442 | 456 | 456 |
| | $z_5$ | 525 | 525 | 525 | 525 | 539 | 539 |
| | $w_3$ | 300 | 300 | 300 (low) | 300 | - | - |
| | $v_2$ | - | 187 | - | 187 | 187 | - |
| | $v_5$ | 514 (low) | 514 (low) | - | - | - | - |
| **Isomeric open specie B** | $b_0$ | 303/305 | 381/383/385 | 329/331 | 406/408/410 | 303/305 | 380/382/384 |
| | $b_1$ | 404/406 | 482/484/486 | - | - | 404/406 | 482/484/486 |
| | $b_3$ | 616/618 (low) | - | - | - | 631/633 (low) | - |
| | $b_4$ | 731/733 | 809/811/813 | - | - | 745/747 (low) | 823/825/827 |
| | $y_2$ | 228 (low) | 228 | 228 | 228 | 228 | 228 |
| | $y_3$ | 342 | 342 | 342 | 342 | 342 | 342 |
| | $y_4$ | 441 (low) | 441 | 441 | 441 | 455 | 455 |
| | $y_5$ | 542 | 542 | 542 | 542 | 556 | 556 |
| | $z_3$ | 325 (low) | 325 (low) | 325 (low) | 325 (low) | 325 | 325 |
| | $z_4$ | 424 (low) | 424 (low) | 424 (low) | 424 (low) | 438 | 438 |
| | $z_5$ | 525 | 525 | 525 | 525 | 539 | 539 |
| | $v_3$ | 283 | - | 283 | - | - | - |
| | $v_4$ | 397 | 397 | 397 | 397 | - | - |
| **Aromatic elimination** | $C_9H_6BrO^+$ | 208/210 | - | - | - | 208/210 | - |
| | $C_9H_5Br_2O^+$ | - | 286/288/290 | - | - | - | 286/288/290 |
| | $C_{11}H_8BrO^+$ | - | - | 234/236 | - | - | - |
| | $C_{11}H_7Br_2O^+$ | - | - | - | 312/314/316 | - | - |

*Fragmentation cleavage sites according to Kalinovskaya et al. 2008. We included $b_0$ as a side chain cleavage; and side chain sub-fragmentation rearrangements. Low less than 5% intensity

**Table S7.** Pairwise comparisons of *Pseudoalteromonas* strains genomes vs type strain genomes in Type (Strain) Genome Server (TSGS)

| Query strain | Subject strain | dDDH* (d0, in %) | C.I.** (d0, in %) | dDDH (d4, in %) | C.I. (d4, in %) | dDDH (d6, in %) | C.I. (d6, in %) | G+C content difference (in %) |
|---|---|---|---|---|---|---|---|---|
| 'Pseudoalteromonas sp. CO133X' (NZ SGPE00000000.1) | 'Pseudoalteromonas sp. CO302Y' (NZ SGPF00000000.1) | 100 | [100.0 - 100.0] | 100 | [100.0 - 100.0] | 100 | [100.0 - 100.0] | 0 |
| 'Pseudoalteromonas sp. CO342X' (NZ SGPH00000000.1) | Pseudoalteromonas maricaloris LMG 19692 | 95.4 | [93.3 - 96.9] | 92.9 | [90.9 - 94.4] | 96.8 | [95.3 - 97.8] | 0.15 |
| 'Pseudoalteromonas sp. CO109Y' (NZ SGPD00000000.1) | Pseudoalteromonas shioyasakiensis JCM 18891 | 92.1 | [89.2 - 94.3] | 87 | [84.4 - 89.2] | 93.7 | [91.5 - 95.4] | 0.01 |
| 'Pseudoalteromonas sp. CO342X' (NZ SGPH00000000.1) | 'Pseudoalteromonas sp. CO348' (NZ SGPI00000000.1) | 82.5 | [78.7 - 85.8] | 79 | [76.0 - 81.7] | 84.9 | [81.7 - 87.6] | 0.06 |
| 'Pseudoalteromonas sp. CO348' (NZ SGPI00000000.1) | Pseudoalteromonas maricaloris LMG 19692 | 79.6 | [75.7 - 83.1] | 78.8 | [75.8 - 81.5] | 82.4 | [79.1 - 85.3] | 0.1 |
| 'Pseudoalteromonas sp. CO325X' (NZ SGPG00000000.1) | Pseudoalteromonas ruthenica LMG 19699 | 87.7 | [84.2 - 90.5] | 78.6 | [75.7 - 81.3] | 89 | [86.1 - 91.3] | 0.28 |
| 'Pseudoalteromonas sp. CO348' (NZ SGPI00000000.1) | Pseudoalteromonas piscicida ATCC 15057 | 81 | [77.1 - 84.4] | 68.8 | [65.8 - 71.6] | 81.6 | [78.3 - 84.6] | 0.02 |
| 'Pseudoalteromonas sp. CO342X' (NZ SGPH00000000.1) | Pseudoalteromonas piscicida ATCC 15057 | 81.3 | [77.4 - 84.7] | 68.7 | [65.7 - 71.5] | 81.8 | [78.5 - 84.8] | 0.04 |
| 'Pseudoalteromonas sp. CO133X' (NZ SGPE00000000.1) | Pseudoalteromonas arabiensis JCM 17292 | 86.4 | [82.8 - 89.4] | 64.1 | [61.1 - 66.9] | 85 | [81.9 - 87.8] | 0.06 |
| 'Pseudoalteromonas sp. CO302Y' (NZ SGPF00000000.1) | Pseudoalteromonas arabiensis JCM 17292 | 86.5 | [82.8 - 89.4] | 64.1 | [61.1 - 66.9] | 85.1 | [81.9 - 87.8] | 0.06 |
| 'Pseudoalteromonas sp. CO109Y' (NZ SGPD00000000.1) | Pseudoalteromonas gelatinilytica NH153 | 85.1 | [81.4 - 88.2] | 52 | [49.3 - 54.7] | 80.5 | [77.1 - 83.5] | 0.12 |
| 'Pseudoalteromonas sp. CO325X' (NZ SGPG00000000.1) | Kangiella profundi FT102 | 12.6 | [10.0 - 15.9] | 36 | [33.6 - 38.5] | 13 | [10.7 - 15.8] | 3.44 |
| 'Pseudoalteromonas sp. CO325X' (NZ SGPG00000000.1) | Salinimonas sediminis KCTC 62440 | 12.8 | [10.1 - 16.1] | 33.1 | [30.7 - 35.7] | 13.2 | [10.9 - 16.0] | 0.96 |
| 'Pseudoalteromonas sp. CO109Y' (NZ SGPD00000000.1) | Salinimonas sediminis KCTC 62440 | 12.6 | [9.9 - 15.8] | 31.5 | [29.1 - 34.0] | 13 | [10.7 - 15.7] | 6.93 |
| 'Pseudoalteromonas sp. CO109Y' (NZ SGPD00000000.1) | 'Pseudoalteromonas sp. CO302Y' (NZ SGPF00000000.1) | 59.2 | [55.5 - 62.7] | 29 | [26.6 - 31.5] | 50.1 | [47.0 - 53.1] | 0.4 |
| 'Pseudoalteromonas sp. CO109Y' (NZ SGPD00000000.1) | 'Pseudoalteromonas sp. CO133X' (NZ SGPE00000000.1) | 59.1 | [55.5 - 62.7] | 29 | [26.6 - 31.5] | 50.1 | [47.0 - 53.1] | 0.4 |
| 'Pseudoalteromonas sp. CO302Y' (NZ SGPF00000000.1) | Pseudoalteromonas gelatinilytica NH153 | 55.1 | [51.6 - 58.6] | 29 | [26.6 - 31.5] | 47.3 | [44.3 - 50.4] | 0.52 |
| 'Pseudoalteromonas sp. CO133X' (NZ SGPE00000000.1) | Pseudoalteromonas gelatinilytica NH153 | 55.1 | [51.6 - 58.6] | 29 | [26.6 - 31.5] | 47.3 | [44.3 - 50.4] | 0.51 |
| 'Pseudoalteromonas sp. CO133X' (NZ SGPE00000000.1) | Pseudoalteromonas shioyasakiensis JCM 18891 | 56.4 | [52.8 - 59.9] | 28.9 | [26.5 - 31.4] | 48.2 | [45.1 - 51.2] | 0.39 |
| 'Pseudoalteromonas sp. CO302Y' (NZ SGPF00000000.1) | Pseudoalteromonas shioyasakiensis JCM 18891 | 56.4 | [52.9 - 59.9] | 28.9 | [26.5 - 31.4] | 48.2 | [45.2 - 51.2] | 0.39 |
| 'Pseudoalteromonas sp. CO109Y' (NZ SGPD00000000.1) | Pseudoalteromonas arabiensis JCM 17292 | 55.4 | [51.9 - 58.9] | 28.3 | [25.9 - 30.8] | 47.2 | [44.2 - 50.2] | 0.34 |
| 'Pseudoalteromonas sp. CO348' (NZ SGPI00000000.1) | Salinimonas sediminis KCTC 62440 | 12.6 | [9.9 - 15.9] | 26.3 | [23.9 - 28.8] | 13 | [10.7 - 15.7] | 5.01 |

| | | | | | | | | |
|---|---|---|---|---|---|---|---|---|
| 'Pseudoalteromonas sp. CO302Y' (NZ SGPF00000000.1) | Salinimonas sediminis KCTC 62440 | 12.6 | [9.9 - 15.9] | 25.1 | [22.8 - 27.6] | 13 | [10.7 - 15.8] | 7.33 |
| 'Pseudoalteromonas sp. CO133X' (NZ SGPE00000000.1) | Salinimonas sediminis KCTC 62440 | 12.6 | [9.9 - 15.9] | 25 | [22.7 - 27.5] | 13 | [10.7 - 15.8] | 7.32 |
| 'Pseudoalteromonas sp. CO109Y' (NZ SGPD00000000.1) | Pseudoalteromonas donghaensis KCTC22219 | 55.4 | [51.9 - 58.9] | 24.6 | [22.3 - 27.1] | 45 | [42.0 - 48.0] | 0.32 |
| 'Pseudoalteromonas sp. CO302Y' (NZ SGPF00000000.1) | Kangiella profundi FT102 | 12.6 | [9.9 - 15.8] | 24.5 | [22.2 - 27.0] | 13 | [10.6 - 15.7] | 2.93 |
| 'Pseudoalteromonas sp. CO133X' (NZ SGPE00000000.1) | Kangiella profundi FT102 | 12.6 | [9.9 - 15.8] | 24.5 | [22.1 - 26.9] | 13 | [10.6 - 15.7] | 2.92 |
| 'Pseudoalteromonas sp. CO109Y' (NZ SGPD00000000.1) | Pseudoalteromonas lipolytica CGMCC 1.8499 | 55 | [51.4 - 58.4] | 24.5 | [22.2 - 27.0] | 44.6 | [41.6 - 47.7] | 0.2 |
| 'Pseudoalteromonas sp. CO342X' (NZ SGPH00000000.1) | Salinimonas sediminis KCTC 62440 | 12.6 | [9.9 - 15.9] | 24.3 | [21.9 - 26.7] | 13 | [10.7 - 15.8] | 4.95 |
| 'Pseudoalteromonas sp. CO342X' (NZ SGPH00000000.1) | Pseudoalteromonas peptidolytica NBRC 101021 | 35.7 | [32.3 - 39.2] | 24 | [21.7 - 26.4] | 31.6 | [28.7 - 34.7] | 0.82 |
| 'Pseudoalteromonas sp. CO133X' (NZ SGPE00000000.1) | Pseudoalteromonas donghaensis KCTC22219 | 48.9 | [45.5 - 52.3] | 24 | [21.7 - 26.4] | 40.5 | [37.5 - 43.5] | 0.72 |
| 'Pseudoalteromonas sp. CO302Y' (NZ SGPF00000000.1) | Pseudoalteromonas donghaensis KCTC22219 | 48.9 | [45.5 - 52.3] | 24 | [21.7 - 26.4] | 40.5 | [37.5 - 43.5] | 0.72 |
| 'Pseudoalteromonas sp. CO348' (NZ SGPI00000000.1) | Pseudoalteromonas peptidolytica NBRC 101021 | 36.3 | [32.9 - 39.8] | 23.9 | [21.6 - 26.3] | 32 | [29.1 - 35.1] | 0.76 |
| 'Pseudoalteromonas sp. CO133X' (NZ SGPE00000000.1) | Pseudoalteromonas lipolytica CGMCC 1.8499 | 48.7 | [45.3 - 52.2] | 23.8 | [21.5 - 26.3] | 40.3 | [37.3 - 43.3] | 0.59 |
| 'Pseudoalteromonas sp. CO302Y' (NZ SGPF00000000.1) | Pseudoalteromonas lipolytica CGMCC 1.8499 | 48.7 | [45.3 - 52.2] | 23.8 | [21.5 - 26.3] | 40.3 | [37.3 - 43.3] | 0.6 |
| 'Pseudoalteromonas sp. CO325X' (NZ SGPG00000000.1) | Vibrio panuliri LBS2 | 12.6 | [9.9 - 15.9] | 23.6 | [21.3 - 26.1] | 13 | [10.7 - 15.8] | 2.14 |
| 'Pseudoalteromonas sp. CO325X' (NZ SGPG00000000.1) | Pseudoalteromonas piratica OCN003 | 13.2 | [10.5 - 16.5] | 22.8 | [20.5 - 25.2] | 13.5 | [11.2 - 16.3] | 7.29 |
| 'Pseudoalteromonas sp. CO348' (NZ SGPI00000000.1) | Pseudoalteromonas piratica OCN003 | 13.3 | [10.6 - 16.7] | 22.5 | [20.3 - 25.0] | 13.7 | [11.3 - 16.5] | 3.24 |
| 'Pseudoalteromonas sp. CO325X' (NZ SGPG00000000.1) | Pseudoalteromonas phenolica KCTC 12086 | 13.4 | [10.7 - 16.8] | 22 | [19.7 - 24.4] | 13.8 | [11.4 - 16.6] | 6.67 |
| 'Pseudoalteromonas sp. CO342X' (NZ SGPH00000000.1) | Pseudoalteromonas piratica OCN003 | 13.4 | [10.6 - 16.7] | 22 | [19.8 - 24.5] | 13.7 | [11.3 - 16.5] | 3.3 |
| 'Pseudoalteromonas sp. CO348' (NZ SGPI00000000.1) | Pseudoalteromonas ruthenica LMG 19699 | 13.4 | [10.6 - 16.7] | 21.8 | [19.5 - 24.2] | 13.7 | [11.3 - 16.5] | 4.33 |
| 'Pseudoalteromonas sp. CO109Y' (NZ SGPD00000000.1) | Kangiella profundi FT102 | 12.6 | [9.9 - 15.8] | 21.8 | [19.6 - 24.3] | 13 | [10.6 - 15.7] | 2.53 |
| 'Pseudoalteromonas sp. CO342X' (NZ SGPH00000000.1) | Pseudoalteromonas ruthenica LMG 19699 | 13.4 | [10.7 - 16.7] | 21.7 | [19.4 - 24.1] | 13.7 | [11.4 - 16.5] | 4.27 |
| 'Pseudoalteromonas sp. CO325X' (NZ SGPG00000000.1) | Pseudoalteromonas citrea NCIMB 1889 | 13.4 | [10.7 - 16.7] | 21.5 | [19.3 - 23.9] | 13.7 | [11.4 - 16.5] | 6.13 |
| 'Pseudoalteromonas sp. CO348' (NZ SGPI00000000.1) | Pseudoalteromonas spongiae JCM 12884 | 13.3 | [10.5 - 16.6] | 21.5 | [19.3 - 24.0] | 13.6 | [11.2 - 16.4] | 2.39 |
| 'Pseudoalteromonas sp. CO325X' (NZ SGPG00000000.1) | Pseudoalteromonas amylolytica JW1 | 13.5 | [10.7 - 16.8] | 21.5 | [19.3 - 24.0] | 13.8 | [11.4 - 16.6] | 3.9 |
| 'Pseudoalteromonas sp. CO342X' (NZ SGPH00000000.1) | Pseudoalteromonas spongiae JCM 12884 | 13.3 | [10.6 - 16.6] | 21.4 | [19.1 - 23.8] | 13.6 | [11.2 - 16.4] | 2.45 |

| | | | | | | | | |
|---|---|---|---|---|---|---|---|---|
| 'Pseudoalteromonas sp. CO325X' (NZ SGPG00000000.1) | Pseudoalteromonas maricaloris LMG 19692 | 13.3 | [10.6 - 16.6] | 21.3 | [19.0 - 23.7] | 13.6 | [11.3 - 16.4] | 4.14 |
| 'Pseudoalteromonas sp. CO325X' (NZ SGPG00000000.1) | 'Pseudoalteromonas sp. CO348' (NZ SGPI00000000.1) | 13.3 | [10.5 - 16.6] | 21.2 | [18.9 - 23.6] | 13.6 | [11.2 - 16.4] | 4.05 |
| 'Pseudoalteromonas sp. CO109Y' (NZ SGPD00000000.1) | Pseudoalteromonas piratica OCN003 | 13.9 | [11.1 - 17.3] | 21.2 | [18.9 - 23.6] | 14.2 | [11.8 - 17.0] | 1.32 |
| 'Pseudoalteromonas sp. CO342X' (NZ SGPH00000000.1) | Pseudoalteromonas agarivorans DSM 14585 | 13.6 | [10.8 - 16.9] | 21.1 | [18.8 - 23.5] | 13.9 | [11.5 - 16.7] | 2.42 |
| 'Pseudoalteromonas sp. CO348' (NZ SGPI00000000.1) | Pseudoalteromonas agarivorans DSM 14585 | 13.6 | [10.9 - 17.0] | 21.1 | [18.8 - 23.5] | 13.9 | [11.5 - 16.7] | 2.36 |
| 'Pseudoalteromonas sp. CO325X' (NZ SGPG00000000.1) | Pseudoalteromonas tetraodonis DSM 9166 | 13.6 | [10.9 - 17.0] | 21.1 | [18.9 - 23.5] | 13.9 | [11.5 - 16.7] | 6.94 |
| 'Pseudoalteromonas sp. CO325X' (NZ SGPG00000000.1) | Pseudoalteromonas issachenkonii KCTC 12958 | 13.6 | [10.8 - 16.9] | 21 | [18.8 - 23.4] | 13.9 | [11.5 - 16.7] | 6.96 |
| 'Pseudoalteromonas sp. CO325X' (NZ SGPG00000000.1) | Pseudoalteromonas piscicida ATCC 15057 | 13.3 | [10.5 - 16.6] | 20.9 | [18.7 - 23.4] | 13.6 | [11.2 - 16.4] | 4.03 |
| 'Pseudoalteromonas sp. CO348' (NZ SGPI00000000.1) | Pseudoalteromonas espejiana ATCC 29659 | 13.6 | [10.9 - 17.0] | 20.9 | [18.6 - 23.3] | 13.9 | [11.5 - 16.7] | 2.9 |
| 'Pseudoalteromonas sp. CO109Y' (NZ SGPD00000000.1) | Pseudoalteromonas tetraodonis DSM 9166 | 19.6 | [16.4 - 23.2] | 20.9 | [18.7 - 23.3] | 19 | [16.3 - 22.0] | 0.97 |
| 'Pseudoalteromonas sp. CO325X' (NZ SGPG00000000.1) | Pseudoalteromonas rubra ATCC 29570 | 13.2 | [10.5 - 16.5] | 20.9 | [18.6 - 23.3] | 13.6 | [11.2 - 16.4] | 0.54 |
| 'Pseudoalteromonas sp. CO348' (NZ SGPI00000000.1) | Pseudoalteromonas phenolica KCTC 12086 | 14.5 | [11.7 - 17.9] | 20.8 | [18.6 - 23.2] | 14.7 | [12.3 - 17.6] | 2.62 |
| 'Pseudoalteromonas sp. CO302Y' (NZ SGPF00000000.1) | Pseudoalteromonas spongiae JCM 12884 | 13.7 | [11.0 - 17.1] | 20.8 | [18.6 - 23.3] | 14 | [11.6 - 16.8] | 0.08 |
| 'Pseudoalteromonas sp. CO325X' (NZ SGPG00000000.1) | Pseudoalteromonas spongiae JCM 12884 | 13.2 | [10.5 - 16.5] | 20.8 | [18.6 - 23.3] | 13.5 | [11.2 - 16.3] | 6.44 |
| 'Pseudoalteromonas sp. CO109Y' (NZ SGPD00000000.1) | Pseudoalteromonas maricaloris LMG 19692 | 14.3 | [11.4 - 17.6] | 20.8 | [18.6 - 23.3] | 14.5 | [12.0 - 17.3] | 1.82 |
| 'Pseudoalteromonas sp. CO133X' (NZ SGPE00000000.1) | Pseudoalteromonas spongiae JCM 12884 | 13.7 | [11.0 - 17.1] | 20.8 | [18.6 - 23.2] | 14 | [11.6 - 16.8] | 0.08 |
| 'Pseudoalteromonas sp. CO342X' (NZ SGPH00000000.1) | Pseudoalteromonas espejiana ATCC 29659 | 13.7 | [10.9 - 17.1] | 20.8 | [18.5 - 23.2] | 14 | [11.6 - 16.8] | 2.96 |
| 'Pseudoalteromonas sp. CO133X' (NZ SGPE00000000.1) | Pseudoalteromonas phenolica KCTC 12086 | 14.5 | [11.7 - 17.9] | 20.8 | [18.5 - 23.2] | 14.7 | [12.3 - 17.6] | 0.31 |
| 'Pseudoalteromonas sp. CO302Y' (NZ SGPF00000000.1) | Pseudoalteromonas phenolica KCTC 12086 | 14.5 | [11.7 - 17.9] | 20.8 | [18.5 - 23.2] | 14.7 | [12.3 - 17.6] | 0.3 |
| 'Pseudoalteromonas sp. CO348' (NZ SGPI00000000.1) | Pseudoalteromonas carrageenovora ATCC 43555 | 13.6 | [10.9 - 17.0] | 20.8 | [18.6 - 23.2] | 13.9 | [11.5 - 16.7] | 3.78 |
| 'Pseudoalteromonas sp. CO109Y' (NZ SGPD00000000.1) | Pseudoalteromonas issachenkonii KCTC 12958 | 19.6 | [16.5 - 23.2] | 20.8 | [18.6 - 23.2] | 19 | [16.3 - 22.0] | 0.99 |
| 'Pseudoalteromonas sp. CO342X' (NZ SGPH00000000.1) | Pseudoalteromonas tetraodonis DSM 9166 | 13.8 | [11.0 - 17.1] | 20.8 | [18.5 - 23.2] | 14.1 | [11.6 - 16.9] | 2.95 |
| 'Pseudoalteromonas sp. CO109Y' (NZ SGPD00000000.1) | Pseudoalteromonas spiralis DSM 16099 | 19.2 | [16.1 - 22.8] | 20.8 | [18.6 - 23.3] | 18.7 | [16.0 - 21.7] | 1.14 |
| 'Pseudoalteromonas sp. CO325X' (NZ SGPG00000000.1) | 'Pseudoalteromonas sp. CO342X' (NZ SGPH00000000.1) | 13.3 | [10.6 - 16.6] | 20.7 | [18.4 - 23.1] | 13.6 | [11.3 - 16.4] | 3.99 |
| 'Pseudoalteromonas sp. CO348' (NZ SGPI00000000.1) | Pseudoalteromonas tetraodonis DSM 9166 | 13.8 | [11.1 - 17.2] | 20.7 | [18.4 - 23.1] | 14.1 | [11.7 - 16.9] | 2.89 |

| | | | | | | | | |
|---|---|---|---|---|---|---|---|---|
| 'Pseudoalteromonas sp. CO109Y' (NZ SGPD00000000.1) | Pseudoalteromonas agarivorans DSM 14585 | 18.9 | [15.8 - 22.5] | 20.7 | [18.5 - 23.1] | 18.4 | [15.8 - 21.4] | 0.45 |
| 'Pseudoalteromonas sp. CO109Y' (NZ SGPD00000000.1) | Pseudoalteromonas atlantica NBRC 103033 | 18.9 | [15.7 - 22.5] | 20.6 | [18.4 - 23.0] | 18.4 | [15.7 - 21.4] | 0.53 |
| 'Pseudoalteromonas sp. CO133X' (NZ SGPE00000000.1) | Pseudoalteromonas ruthenica LMG 19699 | 13.9 | [11.1 - 17.2] | 20.6 | [18.4 - 23.1] | 14.1 | [11.7 - 17.0] | 6.64 |
| 'Pseudoalteromonas sp. CO302Y' (NZ SGPF00000000.1) | Pseudoalteromonas luteoviolacea DSM 6061 | 13.6 | [10.8 - 16.9] | 20.6 | [18.4 - 23.0] | 13.9 | [11.5 - 16.7] | 0.89 |
| 'Pseudoalteromonas sp. CO325X' (NZ SGPG00000000.1) | Pseudoalteromonas luteoviolacea DSM 6061 | 13.2 | [10.5 - 16.5] | 20.6 | [18.3 - 23.0] | 13.6 | [11.2 - 16.3] | 5.48 |
| 'Pseudoalteromonas sp. CO133X' (NZ SGPE00000000.1) | Pseudoalteromonas luteoviolacea DSM 6061 | 13.6 | [10.8 - 16.9] | 20.6 | [18.4 - 23.0] | 13.9 | [11.5 - 16.7] | 0.88 |
| 'Pseudoalteromonas sp. CO302Y' (NZ SGPF00000000.1) | Pseudoalteromonas ruthenica LMG 19699 | 13.9 | [11.1 - 17.2] | 20.6 | [18.4 - 23.1] | 14.1 | [11.7 - 17.0] | 6.64 |
| 'Pseudoalteromonas sp. CO342X' (NZ SGPH00000000.1) | Pseudoalteromonas issachenkonii KCTC 12958 | 13.8 | [11.0 - 17.2] | 20.6 | [18.3 - 23.0] | 14.1 | [11.7 - 16.9] | 2.97 |
| 'Pseudoalteromonas sp. CO342X' (NZ SGPH00000000.1) | Pseudoalteromonas phenolica KCTC 12086 | 14.6 | [11.8 - 18.0] | 20.5 | [18.3 - 22.9] | 14.8 | [12.3 - 17.7] | 2.68 |
| 'Pseudoalteromonas sp. CO109Y' (NZ SGPD00000000.1) | Pseudoalteromonas telluritireducens DSM 16098 | 18.9 | [15.8 - 22.5] | 20.5 | [18.2 - 22.9] | 18.4 | [15.7 - 21.4] | 0.37 |
| 'Pseudoalteromonas sp. CO325X' (NZ SGPG00000000.1) | Pseudoalteromonas spiralis DSM 16099 | 13.6 | [10.9 - 17.0] | 20.5 | [18.3 - 22.9] | 13.9 | [11.5 - 16.7] | 7.1 |
| 'Pseudoalteromonas sp. CO109Y' (NZ SGPD00000000.1) | Pseudoalteromonas carrageenovora ATCC 43555 | 19.2 | [16.1 - 22.8] | 20.5 | [18.3 - 22.9] | 18.6 | [16.0 - 21.7] | 1.87 |
| 'Pseudoalteromonas sp. CO302Y' (NZ SGPF00000000.1) | Pseudoalteromonas piratica OCN003 | 13.9 | [11.1 - 17.2] | 20.5 | [18.3 - 22.9] | 14.1 | [11.7 - 17.0] | 0.92 |
| 'Pseudoalteromonas sp. CO109Y' (NZ SGPD00000000.1) | Pseudoalteromonas haloplanktis ATCC 14393 | 21.9 | [18.6 - 25.5] | 20.5 | [18.3 - 23.0] | 20.7 | [18.0 - 23.8] | 0.44 |
| 'Pseudoalteromonas sp. CO133X' (NZ SGPE00000000.1) | Pseudoalteromonas piratica OCN003 | 13.9 | [11.1 - 17.2] | 20.5 | [18.3 - 22.9] | 14.1 | [11.7 - 17.0] | 0.92 |
| 'Pseudoalteromonas sp. CO302Y' (NZ SGPF00000000.1) | Pseudoalteromonas rubra ATCC 29570 | 13.3 | [10.6 - 16.7] | 20.4 | [18.2 - 22.8] | 13.7 | [11.3 - 16.5] | 6.91 |
| 'Pseudoalteromonas sp. CO348' (NZ SGPI00000000.1) | Pseudoalteromonas issachenkonii KCTC 12958 | 13.8 | [11.0 - 17.2] | 20.4 | [18.2 - 22.9] | 14.1 | [11.7 - 16.9] | 2.91 |
| 'Pseudoalteromonas sp. CO133X' (NZ SGPE00000000.1) | Pseudoalteromonas rubra ATCC 29570 | 13.3 | [10.6 - 16.7] | 20.4 | [18.2 - 22.8] | 13.7 | [11.3 - 16.5] | 6.9 |
| 'Pseudoalteromonas sp. CO342X' (NZ SGPH00000000.1) | Pseudoalteromonas spiralis DSM 16099 | 13.8 | [11.0 - 17.1] | 20.4 | [18.2 - 22.8] | 14 | [11.6 - 16.9] | 3.11 |
| 'Pseudoalteromonas sp. CO325X' (NZ SGPG00000000.1) | Pseudoalteromonas byunsanensis JCM 12483 | 13.6 | [10.8 - 16.9] | 20.4 | [18.2 - 22.8] | 13.9 | [11.5 - 16.7] | 4.77 |
| 'Pseudoalteromonas sp. CO133X' (NZ SGPE00000000.1) | Vibrio panuliri LBS2 | 12.7 | [10.1 - 16.0] | 20.4 | [18.2 - 22.8] | 13.1 | [10.8 - 15.9] | 4.22 |
| 'Pseudoalteromonas sp. CO302Y' (NZ SGPF00000000.1) | Vibrio panuliri LBS2 | 12.7 | [10.1 - 16.0] | 20.4 | [18.2 - 22.8] | 13.1 | [10.8 - 15.9] | 4.23 |
| 'Pseudoalteromonas sp. CO109Y' (NZ SGPD00000000.1) | Pseudoalteromonas phenolica KCTC 12086 | 14.7 | [11.8 - 18.1] | 20.4 | [18.1 - 22.8] | 14.8 | [12.4 - 17.7] | 0.7 |
| 'Pseudoalteromonas sp. CO109Y' (NZ SGPD00000000.1) | Pseudoalteromonas espejiana ATCC 29659 | 19.8 | [16.6 - 23.4] | 20.4 | [18.2 - 22.8] | 19 | [16.4 - 22.1] | 0.99 |
| 'Pseudoalteromonas sp. CO348' (NZ SGPI00000000.1) | Pseudoalteromonas telluritireducens DSM 16098 | 13.6 | [10.8 - 16.9] | 20.4 | [18.2 - 22.8] | 13.9 | [11.5 - 16.7] | 2.28 |

| | | | | | | | | |
|---|---|---|---|---|---|---|---|---|
| 'Pseudoalteromonas sp. CO325X' (NZ SGPG00000000.1) | Pseudoalteromonas peptidolytica NBRC 101021 | 13.3 | [10.6 - 16.6] | 20.4 | [18.2 - 22.8] | 13.7 | [11.3 - 16.4] | 4.81 |
| 'Pseudoalteromonas sp. CO109Y' (NZ SGPD00000000.1) | Pseudoalteromonas neustonica PAMC 28425 | 20.1 | [16.9 - 23.7] | 20.4 | [18.2 - 22.8] | 19.3 | [16.6 - 22.4] | 1.56 |
| 'Pseudoalteromonas sp. CO109Y' (NZ SGPD00000000.1) | Pseudoalteromonas prydzensis DSM 14232 | 21.6 | [18.4 - 25.2] | 20.3 | [18.1 - 22.7] | 20.5 | [17.7 - 23.5] | 0.13 |
| 'Pseudoalteromonas sp. CO302Y' (NZ SGPF00000000.1) | Pseudoalteromonas tetraodonis DSM 9166 | 19.2 | [16.0 - 22.8] | 20.3 | [18.1 - 22.7] | 18.6 | [15.9 - 21.6] | 0.58 |
| 'Pseudoalteromonas sp. CO133X' (NZ SGPE00000000.1) | 'Pseudoalteromonas sp. CO348' (NZ SGPI00000000.1) | 14.1 | [11.3 - 17.4] | 20.3 | [18.1 - 22.7] | 14.3 | [11.9 - 17.1] | 2.31 |
| 'Pseudoalteromonas sp. CO302Y' (NZ SGPF00000000.1) | 'Pseudoalteromonas sp. CO348' (NZ SGPI00000000.1) | 14.1 | [11.3 - 17.4] | 20.3 | [18.1 - 22.7] | 14.3 | [11.9 - 17.1] | 2.32 |
| 'Pseudoalteromonas sp. CO133X' (NZ SGPE00000000.1) | Pseudoalteromonas agarivorans DSM 14585 | 19.5 | [16.3 - 23.1] | 20.3 | [18.1 - 22.7] | 18.8 | [16.1 - 21.8] | 0.05 |
| 'Pseudoalteromonas sp. CO109Y' (NZ SGPD00000000.1) | Pseudoalteromonas peptidolytica NBRC 101021 | 13.8 | [11.0 - 17.1] | 20.3 | [18.1 - 22.7] | 14 | [11.6 - 16.9] | 1.15 |
| 'Pseudoalteromonas sp. CO302Y' (NZ SGPF00000000.1) | Pseudoalteromonas agarivorans DSM 14585 | 19.5 | [16.3 - 23.1] | 20.3 | [18.1 - 22.7] | 18.8 | [16.1 - 21.8] | 0.05 |
| 'Pseudoalteromonas sp. CO133X' (NZ SGPE00000000.1) | Pseudoalteromonas tetraodonis DSM 9166 | 19.2 | [16.0 - 22.8] | 20.3 | [18.1 - 22.7] | 18.6 | [15.9 - 21.6] | 0.58 |
| 'Pseudoalteromonas sp. CO348' (NZ SGPI00000000.1) | Pseudoalteromonas donghaensis KCTC22219 | 14.1 | [11.3 - 17.4] | 20.3 | [18.1 - 22.7] | 14.3 | [11.9 - 17.1] | 1.6 |
| 'Pseudoalteromonas sp. CO302Y' (NZ SGPF00000000.1) | Pseudoalteromonas espejiana ATCC 29659 | 19.9 | [16.7 - 23.5] | 20.3 | [18.1 - 22.7] | 19.1 | [16.4 - 22.2] | 0.59 |
| 'Pseudoalteromonas sp. CO133X' (NZ SGPE00000000.1) | Pseudoalteromonas espejiana ATCC 29659 | 19.9 | [16.7 - 23.5] | 20.3 | [18.1 - 22.7] | 19.1 | [16.4 - 22.1] | 0.59 |
| 'Pseudoalteromonas sp. CO302Y' (NZ SGPF00000000.1) | Pseudoalteromonas piscicida ATCC 15057 | 14 | [11.2 - 17.4] | 20.2 | [18.0 - 22.6] | 14.3 | [11.9 - 17.1] | 2.34 |
| 'Pseudoalteromonas sp. CO133X' (NZ SGPE00000000.1) | 'Pseudoalteromonas sp. CO342X' (NZ SGPH00000000.1) | 14.1 | [11.3 - 17.4] | 20.2 | [18.0 - 22.6] | 14.3 | [11.9 - 17.1] | 2.37 |
| 'Pseudoalteromonas sp. CO302Y' (NZ SGPF00000000.1) | Pseudoalteromonas carrageenovora ATCC 43555 | 19.7 | [16.5 - 23.3] | 20.2 | [18.0 - 22.6] | 19 | [16.3 - 22.0] | 1.47 |
| 'Pseudoalteromonas sp. CO342X' (NZ SGPH00000000.1) | Pseudoalteromonas donghaensis KCTC22219 | 14.1 | [11.3 - 17.5] | 20.2 | [18.0 - 22.7] | 14.4 | [11.9 - 17.2] | 1.66 |
| 'Pseudoalteromonas sp. CO133X' (NZ SGPE00000000.1) | Pseudoalteromonas issachenkonii KCTC 12958 | 19.1 | [16.0 - 22.7] | 20.2 | [18.0 - 22.6] | 18.5 | [15.9 - 21.5] | 0.6 |
| 'Pseudoalteromonas sp. CO133X' (NZ SGPE00000000.1) | Pseudoalteromonas carrageenovora ATCC 43555 | 19.6 | [16.5 - 23.3] | 20.2 | [18.0 - 22.6] | 18.9 | [16.3 - 22.0] | 1.47 |
| 'Pseudoalteromonas sp. CO302Y' (NZ SGPF00000000.1) | Pseudoalteromonas maricaloris LMG 19692 | 14 | [11.2 - 17.4] | 20.2 | [18.0 - 22.7] | 14.3 | [11.9 - 17.1] | 2.22 |
| 'Pseudoalteromonas sp. CO342X' (NZ SGPH00000000.1) | Pseudoalteromonas carrageenovora ATCC 43555 | 13.8 | [11.0 - 17.1] | 20.2 | [18.0 - 22.6] | 14 | [11.6 - 16.8] | 3.84 |
| 'Pseudoalteromonas sp. CO325X' (NZ SGPG00000000.1) | Pseudoalteromonas agarivorans DSM 14585 | 13.8 | [11.0 - 17.1] | 20.2 | [18.0 - 22.6] | 14 | [11.6 - 16.9] | 6.41 |
| 'Pseudoalteromonas sp. CO133X' (NZ SGPE00000000.1) | Pseudoalteromonas maricaloris LMG 19692 | 14 | [11.2 - 17.4] | 20.2 | [18.0 - 22.7] | 14.3 | [11.9 - 17.1] | 2.22 |
| 'Pseudoalteromonas sp. CO302Y' (NZ SGPF00000000.1) | Pseudoalteromonas issachenkonii KCTC 12958 | 19.1 | [16.0 - 22.7] | 20.2 | [18.0 - 22.6] | 18.5 | [15.9 - 21.5] | 0.59 |
| 'Pseudoalteromonas sp. CO133X' (NZ SGPE00000000.1) | Pseudoalteromonas piscicida ATCC 15057 | 14 | [11.2 - 17.4] | 20.2 | [18.0 - 22.6] | 14.3 | [11.9 - 17.1] | 2.34 |

| | | | | | | | | |
|---|---|---|---|---|---|---|---|---|
| 'Pseudoalteromonas sp. CO348' (NZ SGPI00000000.1) | Pseudoalteromonas spiralis DSM 16099 | 13.7 | [11.0 - 17.1] | 20.2 | [18.0 - 22.6] | 14 | [11.6 - 16.8] | 3.05 |
| 'Pseudoalteromonas sp. CO348' (NZ SGPI00000000.1) | Pseudoalteromonas gelatinilytica NH153 | 14 | [11.2 - 17.4] | 20.1 | [17.9 - 22.5] | 14.3 | [11.8 - 17.1] | 1.8 |
| 'Pseudoalteromonas sp. CO342X' (NZ SGPH00000000.1) | Pseudoalteromonas gelatinilytica NH153 | 14.1 | [11.3 - 17.5] | 20.1 | [17.8 - 22.5] | 14.3 | [11.9 - 17.1] | 1.86 |
| 'Pseudoalteromonas sp. CO133X' (NZ SGPE00000000.1) | Pseudoalteromonas spiralis DSM 16099 | 19 | [15.9 - 22.6] | 20.1 | [17.9 - 22.5] | 18.4 | [15.8 - 21.4] | 0.74 |
| 'Pseudoalteromonas sp. CO348' (NZ SGPI00000000.1) | Pseudoalteromonas byunsanensis JCM 12483 | 14.8 | [11.9 - 18.2] | 20.1 | [17.9 - 22.5] | 14.9 | [12.5 - 17.8] | 0.73 |
| 'Pseudoalteromonas sp. CO109Y' (NZ SGPD00000000.1) | Pseudoalteromonas arctica DSM 18437 | 19 | [15.9 - 22.6] | 20.1 | [17.9 - 22.5] | 18.4 | [15.7 - 21.4] | 2.24 |
| 'Pseudoalteromonas sp. CO342X' (NZ SGPH00000000.1) | Pseudoalteromonas telluritireducens DSM 16098 | 13.6 | [10.8 - 16.9] | 20.1 | [17.9 - 22.5] | 13.9 | [11.5 - 16.7] | 2.34 |
| 'Pseudoalteromonas sp. CO302Y' (NZ SGPF00000000.1) | Pseudoalteromonas spiralis DSM 16099 | 19 | [15.9 - 22.6] | 20.1 | [17.9 - 22.5] | 18.4 | [15.8 - 21.4] | 0.74 |
| 'Pseudoalteromonas sp. CO109Y' (NZ SGPD00000000.1) | Vibrio panuliri LBS2 | 12.7 | [10.0 - 16.0] | 20.1 | [17.9 - 22.6] | 13.1 | [10.8 - 15.9] | 3.83 |
| 'Pseudoalteromonas sp. CO342X' (NZ SGPH00000000.1) | Kangiella profundi FT102 | 12.6 | [9.9 - 15.9] | 20.1 | [17.9 - 22.5] | 13 | [10.7 - 15.7] | 0.55 |
| 'Pseudoalteromonas sp. CO302Y' (NZ SGPF00000000.1) | 'Pseudoalteromonas sp. CO342X' (NZ SGPH00000000.1) | 14.1 | [11.3 - 17.4] | 20.1 | [17.9 - 22.6] | 14.3 | [11.9 - 17.1] | 2.37 |
| 'Pseudoalteromonas sp. CO133X' (NZ SGPE00000000.1) | Pseudoalteromonas atlantica NBRC 103033 | 19.2 | [16.0 - 22.8] | 20.1 | [17.9 - 22.5] | 18.6 | [15.9 - 21.6] | 0.13 |
| 'Pseudoalteromonas sp. CO109Y' (NZ SGPD00000000.1) | Pseudoalteromonas rubra ATCC 29570 | 13.5 | [10.7 - 16.8] | 20.1 | [17.9 - 22.5] | 13.8 | [11.4 - 16.6] | 6.51 |
| 'Pseudoalteromonas sp. CO325X' (NZ SGPG00000000.1) | Pseudoalteromonas espejiana ATCC 29659 | 13.7 | [10.9 - 17.0] | 20.1 | [17.9 - 22.6] | 14 | [11.6 - 16.8] | 6.95 |
| 'Pseudoalteromonas sp. CO302Y' (NZ SGPF00000000.1) | Pseudoalteromonas atlantica NBRC 103033 | 19.2 | [16.0 - 22.8] | 20.1 | [17.9 - 22.5] | 18.6 | [15.9 - 21.6] | 0.13 |
| 'Pseudoalteromonas sp. CO109Y' (NZ SGPD00000000.1) | Pseudoalteromonas marina mano4 | 19.2 | [16.1 - 22.8] | 20.1 | [17.9 - 22.5] | 18.6 | [15.9 - 21.6] | 1.63 |
| 'Pseudoalteromonas sp. CO302Y' (NZ SGPF00000000.1) | Pseudoalteromonas citrea NCIMB 1889 | 14.1 | [11.3 - 17.4] | 20 | [17.8 - 22.4] | 14.3 | [11.9 - 17.1] | 0.24 |
| 'Pseudoalteromonas sp. CO348' (NZ SGPI00000000.1) | Kangiella profundi FT102 | 12.6 | [9.9 - 15.9] | 20 | [17.7 - 22.4] | 13 | [10.7 - 15.7] | 0.61 |
| 'Pseudoalteromonas sp. CO302Y' (NZ SGPF00000000.1) | Pseudoalteromonas arctica DSM 18437 | 18.8 | [15.7 - 22.4] | 20 | [17.7 - 22.4] | 18.2 | [15.6 - 21.2] | 1.84 |
| 'Pseudoalteromonas sp. CO133X' (NZ SGPE00000000.1) | Pseudoalteromonas citrea NCIMB 1889 | 14.1 | [11.3 - 17.4] | 20 | [17.8 - 22.4] | 14.3 | [11.9 - 17.1] | 0.24 |
| 'Pseudoalteromonas sp. CO342X' (NZ SGPH00000000.1) | Pseudoalteromonas luteoviolacea DSM 6061 | 14.3 | [11.5 - 17.7] | 20 | [17.8 - 22.4] | 14.5 | [12.1 - 17.3] | 1.49 |
| 'Pseudoalteromonas sp. CO109Y' (NZ SGPD00000000.1) | Pseudoalteromonas ruthenica LMG 19699 | 14 | [11.2 - 17.3] | 20 | [17.8 - 22.4] | 14.2 | [11.8 - 17.0] | 6.24 |
| 'Pseudoalteromonas sp. CO302Y' (NZ SGPF00000000.1) | Pseudoalteromonas telluritireducens DSM 16098 | 19.9 | [16.7 - 23.5] | 20 | [17.8 - 22.4] | 19.1 | [16.4 - 22.1] | 0.03 |
| 'Pseudoalteromonas sp. CO342X' (NZ SGPH00000000.1) | Pseudoalteromonas byunsanensis JCM 12483 | 14.7 | [11.9 - 18.2] | 20 | [17.7 - 22.4] | 14.9 | [12.4 - 17.7] | 0.78 |
| 'Pseudoalteromonas sp. CO109Y' (NZ SGPD00000000.1) | Pseudoalteromonas luteoviolacea DSM 6061 | 13.7 | [11.0 - 17.1] | 20 | [17.8 - 22.4] | 14 | [11.6 - 16.8] | 0.49 |

| | | | | | | | | |
|---|---|---|---|---|---|---|---|---|
| 'Pseudoalteromonas sp. CO325X' (NZ SGPG00000000.1) | Pseudoalteromonas haloplanktis ATCC 14393 | 13.4 | [10.7 - 16.8] | 20 | [17.8 - 22.4] | 13.7 | [11.4 - 16.5] | 6.41 |
| 'Pseudoalteromonas sp. CO325X' (NZ SGPG00000000.1) | Pseudoalteromonas donghaensis KCTC22219 | 13.9 | [11.1 - 17.3] | 20 | [17.8 - 22.4] | 14.1 | [11.7 - 17.0] | 5.65 |
| 'Pseudoalteromonas sp. CO302Y' (NZ SGPF00000000.1) | Pseudoalteromonas neustonica PAMC 28425 | 20.3 | [17.1 - 23.9] | 20 | [17.8 - 22.4] | 19.4 | [16.7 - 22.4] | 1.16 |
| 'Pseudoalteromonas sp. CO133X' (NZ SGPE00000000.1) | Pseudoalteromonas neustonica PAMC 28425 | 20.3 | [17.1 - 23.9] | 20 | [17.8 - 22.4] | 19.4 | [16.7 - 22.4] | 1.16 |
| 'Pseudoalteromonas sp. CO348' (NZ SGPI00000000.1) | Pseudoalteromonas arabiensis JCM 17292 | 14 | [11.2 - 17.3] | 20 | [17.8 - 22.4] | 14.2 | [11.8 - 17.0] | 2.25 |
| 'Pseudoalteromonas sp. CO109Y' (NZ SGPD00000000.1) | Pseudoalteromonas undina NCIMB 2128 | 19.4 | [16.2 - 23.0] | 20 | [17.8 - 22.4] | 18.7 | [16.0 - 21.7] | 1.34 |
| 'Pseudoalteromonas sp. CO133X' (NZ SGPE00000000.1) | Pseudoalteromonas telluritireducens DSM 16098 | 19.9 | [16.7 - 23.5] | 20 | [17.8 - 22.4] | 19.1 | [16.4 - 22.1] | 0.03 |
| 'Pseudoalteromonas sp. CO348' (NZ SGPI00000000.1) | Pseudoalteromonas undina NCIMB 2128 | 13.6 | [10.8 - 16.9] | 20 | [17.8 - 22.4] | 13.9 | [11.5 - 16.7] | 3.26 |
| 'Pseudoalteromonas sp. CO133X' (NZ SGPE00000000.1) | Pseudoalteromonas arctica DSM 18437 | 18.8 | [15.7 - 22.4] | 19.9 | [17.7 - 22.4] | 18.2 | [15.6 - 21.2] | 1.84 |
| 'Pseudoalteromonas sp. CO109Y' (NZ SGPD00000000.1) | Pseudoalteromonas spongiae JCM 12884 | 14 | [11.2 - 17.3] | 19.9 | [17.7 - 22.4] | 14.2 | [11.8 - 17.0] | 0.48 |
| 'Pseudoalteromonas sp. CO342X' (NZ SGPH00000000.1) | Pseudoalteromonas arabiensis JCM 17292 | 14 | [11.2 - 17.3] | 19.9 | [17.7 - 22.4] | 14.2 | [11.8 - 17.0] | 2.31 |
| 'Pseudoalteromonas sp. CO348' (NZ SGPI00000000.1) | Pseudoalteromonas rubra ATCC 29570 | 14.5 | [11.6 - 17.8] | 19.9 | [17.7 - 22.3] | 14.6 | [12.2 - 17.5] | 4.59 |
| 'Pseudoalteromonas sp. CO348' (NZ SGPI00000000.1) | Pseudoalteromonas luteoviolacea DSM 6061 | 14.2 | [11.4 - 17.6] | 19.9 | [17.7 - 22.3] | 14.4 | [12.0 - 17.3] | 1.43 |
| 'Pseudoalteromonas sp. CO348' (NZ SGPI00000000.1) | Vibrio panuliri LBS2 | 12.8 | [10.1 - 16.0] | 19.9 | [17.7 - 22.3] | 13.1 | [10.8 - 15.9] | 1.91 |
| 'Pseudoalteromonas sp. CO133X' (NZ SGPE00000000.1) | 'Pseudoalteromonas sp. CO325X' (NZ SGPG00000000.1) | 13.9 | [11.1 - 17.2] | 19.9 | [17.7 - 22.3] | 14.1 | [11.7 - 16.9] | 6.36 |
| 'Pseudoalteromonas sp. CO133X' (NZ SGPE00000000.1) | Pseudoalteromonas haloplanktis ATCC 14393 | 21.6 | [18.4 - 25.2] | 19.9 | [17.7 - 22.3] | 20.4 | [17.7 - 23.5] | 0.04 |
| 'Pseudoalteromonas sp. CO302Y' (NZ SGPF00000000.1) | Pseudoalteromonas amylolytica JW1 | 14.3 | [11.5 - 17.7] | 19.9 | [17.7 - 22.3] | 14.5 | [12.1 - 17.3] | 2.46 |
| 'Pseudoalteromonas sp. CO302Y' (NZ SGPF00000000.1) | 'Pseudoalteromonas sp. CO325X' (NZ SGPG00000000.1) | 13.9 | [11.1 - 17.2] | 19.9 | [17.7 - 22.3] | 14.1 | [11.7 - 16.9] | 6.36 |
| 'Pseudoalteromonas sp. CO302Y' (NZ SGPF00000000.1) | Pseudoalteromonas haloplanktis ATCC 14393 | 21.6 | [18.4 - 25.2] | 19.9 | [17.7 - 22.3] | 20.4 | [17.7 - 23.5] | 0.04 |
| 'Pseudoalteromonas sp. CO302Y' (NZ SGPF00000000.1) | Pseudoalteromonas undina NCIMB 2128 | 19.7 | [16.5 - 23.3] | 19.9 | [17.7 - 22.3] | 18.9 | [16.3 - 22.0] | 0.94 |
| 'Pseudoalteromonas sp. CO133X' (NZ SGPE00000000.1) | Pseudoalteromonas undina NCIMB 2128 | 19.7 | [16.5 - 23.3] | 19.9 | [17.7 - 22.3] | 18.9 | [16.3 - 22.0] | 0.94 |
| 'Pseudoalteromonas sp. CO133X' (NZ SGPE00000000.1) | Pseudoalteromonas amylolytica JW1 | 14.3 | [11.5 - 17.7] | 19.9 | [17.7 - 22.3] | 14.5 | [12.1 - 17.3] | 2.46 |
| 'Pseudoalteromonas sp. CO325X' (NZ SGPG00000000.1) | Pseudoalteromonas arabiensis JCM 17292 | 13.8 | [11.0 - 17.2] | 19.9 | [17.7 - 22.3] | 14.1 | [11.7 - 16.9] | 6.3 |
| 'Pseudoalteromonas sp. CO342X' (NZ SGPH00000000.1) | Pseudoalteromonas amylolytica JW1 | 14.9 | [12.0 - 18.3] | 19.9 | [17.7 - 22.3] | 15 | [12.5 - 17.9] | 0.09 |
| 'Pseudoalteromonas sp. CO109Y' (NZ SGPD00000000.1) | Pseudoalteromonas amylolytica JW1 | 14.5 | [11.6 - 17.9] | 19.8 | [17.6 - 22.2] | 14.6 | [12.2 - 17.5] | 2.06 |

| | | | | | | | | |
|---|---|---|---|---|---|---|---|---|
| 'Pseudoalteromonas sp. CO342X' (NZ SGPH00000000.1) | Pseudoalteromonas undina NCIMB 2128 | 13.6 | [10.9 - 17.0] | 19.8 | [17.6 - 22.2] | 13.9 | [11.5 - 16.7] | 3.31 |
| 'Pseudoalteromonas sp. CO109Y' (NZ SGPD00000000.1) | Pseudoalteromonas byunsanensis JCM 12483 | 14.4 | [11.5 - 17.7] | 19.8 | [17.6 - 22.2] | 14.5 | [12.1 - 17.4] | 1.19 |
| 'Pseudoalteromonas sp. CO325X' (NZ SGPG00000000.1) | Pseudoalteromonas shioyasakiensis JCM 18891 | 13.8 | [11.0 - 17.2] | 19.8 | [17.6 - 22.3] | 14.1 | [11.7 - 16.9] | 5.97 |
| 'Pseudoalteromonas sp. CO109Y' (NZ SGPD00000000.1) | 'Pseudoalteromonas sp. CO348' (NZ SGPI00000000.1) | 14.1 | [11.3 - 17.5] | 19.8 | [17.6 - 22.2] | 14.3 | [11.9 - 17.1] | 1.92 |
| 'Pseudoalteromonas sp. CO325X' (NZ SGPG00000000.1) | Pseudoalteromonas neustonica PAMC 28425 | 13.4 | [10.6 - 16.7] | 19.8 | [17.6 - 22.2] | 13.7 | [11.3 - 16.5] | 7.52 |
| 'Pseudoalteromonas sp. CO133X' (NZ SGPE00000000.1) | Pseudoalteromonas prydzensis DSM 14232 | 22.4 | [19.1 - 26.0] | 19.8 | [17.6 - 22.2] | 21 | [18.2 - 24.1] | 0.27 |
| 'Pseudoalteromonas sp. CO302Y' (NZ SGPF00000000.1) | Pseudoalteromonas prydzensis DSM 14232 | 22.4 | [19.1 - 26.0] | 19.8 | [17.6 - 22.2] | 21 | [18.2 - 24.1] | 0.27 |
| 'Pseudoalteromonas sp. CO302Y' (NZ SGPF00000000.1) | Pseudoalteromonas denitrificans DSM 6059 | 13.1 | [10.4 - 16.4] | 19.7 | [17.5 - 22.1] | 13.5 | [11.1 - 16.2] | 6.16 |
| 'Pseudoalteromonas sp. CO348' (NZ SGPI00000000.1) | Pseudoalteromonas lipolytica CGMCC 1.8499 | 14 | [11.2 - 17.4] | 19.7 | [17.5 - 22.1] | 14.2 | [11.8 - 17.1] | 1.72 |
| 'Pseudoalteromonas sp. CO348' (NZ SGPI00000000.1) | Pseudoalteromonas denitrificans DSM 6059 | 12.8 | [10.1 - 16.1] | 19.7 | [17.5 - 22.1] | 13.2 | [10.9 - 16.0] | 8.48 |
| 'Pseudoalteromonas sp. CO348' (NZ SGPI00000000.1) | Pseudoalteromonas amylolytica JW1 | 14.9 | [12.0 - 18.3] | 19.7 | [17.5 - 22.1] | 15 | [12.6 - 17.9] | 0.15 |
| 'Pseudoalteromonas sp. CO325X' (NZ SGPG00000000.1) | Pseudoalteromonas undina NCIMB 2128 | 13.5 | [10.7 - 16.8] | 19.7 | [17.5 - 22.1] | 13.8 | [11.4 - 16.6] | 7.3 |
| 'Pseudoalteromonas sp. CO325X' (NZ SGPG00000000.1) | Pseudoalteromonas gelatinilytica NH153 | 13.9 | [11.1 - 17.3] | 19.7 | [17.5 - 22.1] | 14.2 | [11.7 - 17.0] | 5.85 |
| 'Pseudoalteromonas sp. CO348' (NZ SGPI00000000.1) | Pseudoalteromonas marina mano4 | 13.6 | [10.8 - 16.9] | 19.7 | [17.5 - 22.1] | 13.9 | [11.5 - 16.7] | 3.55 |
| 'Pseudoalteromonas sp. CO348' (NZ SGPI00000000.1) | Pseudoalteromonas shioyasakiensis JCM 18891 | 14.1 | [11.3 - 17.5] | 19.7 | [17.5 - 22.1] | 14.3 | [11.9 - 17.1] | 1.92 |
| 'Pseudoalteromonas sp. CO342X' (NZ SGPH00000000.1) | Pseudoalteromonas atlantica NBRC 103033 | 13.6 | [10.8 - 16.9] | 19.7 | [17.5 - 22.1] | 13.9 | [11.5 - 16.7] | 2.5 |
| 'Pseudoalteromonas sp. CO133X' (NZ SGPE00000000.1) | Pseudoalteromonas denitrificans DSM 6059 | 13.1 | [10.4 - 16.4] | 19.7 | [17.5 - 22.1] | 13.5 | [11.1 - 16.2] | 6.16 |
| 'Pseudoalteromonas sp. CO342X' (NZ SGPH00000000.1) | Pseudoalteromonas shioyasakiensis JCM 18891 | 14.1 | [11.3 - 17.5] | 19.7 | [17.5 - 22.1] | 14.3 | [11.9 - 17.2] | 1.98 |
| 'Pseudoalteromonas sp. CO109Y' (NZ SGPD00000000.1) | 'Pseudoalteromonas sp. CO342X' (NZ SGPH00000000.1) | 14.1 | [11.3 - 17.5] | 19.7 | [17.5 - 22.1] | 14.3 | [11.9 - 17.2] | 1.98 |
| 'Pseudoalteromonas sp. CO348' (NZ SGPI00000000.1) | Pseudoalteromonas atlantica NBRC 103033 | 13.6 | [10.8 - 16.9] | 19.7 | [17.5 - 22.1] | 13.9 | [11.5 - 16.7] | 2.44 |
| 'Pseudoalteromonas sp. CO342X' (NZ SGPH00000000.1) | Pseudoalteromonas citrea NCIMB 1889 | 14.5 | [11.6 - 17.8] | 19.6 | [17.4 - 22.0] | 14.6 | [12.2 - 17.5] | 2.14 |
| 'Pseudoalteromonas sp. CO109Y' (NZ SGPD00000000.1) | Pseudoalteromonas piscicida ATCC 15057 | 14.1 | [11.3 - 17.5] | 19.6 | [17.4 - 22.0] | 14.3 | [11.9 - 17.1] | 1.94 |
| 'Pseudoalteromonas sp. CO325X' (NZ SGPG00000000.1) | Pseudoalteromonas carrageenovora ATCC 43555 | 13.8 | [11.0 - 17.2] | 19.6 | [17.4 - 22.0] | 14.1 | [11.6 - 16.9] | 7.83 |
| 'Pseudoalteromonas sp. CO302Y' (NZ SGPF00000000.1) | Pseudoalteromonas byunsanensis JCM 12483 | 14.4 | [11.5 - 17.8] | 19.6 | [17.4 - 22.0] | 14.5 | [12.1 - 17.4] | 1.59 |
| 'Pseudoalteromonas sp. CO342X' (NZ SGPH00000000.1) | Pseudoalteromonas lipolytica CGMCC 1.8499 | 14 | [11.2 - 17.4] | 19.6 | [17.4 - 22.0] | 14.3 | [11.8 - 17.1] | 1.78 |

| | | | | | | | | |
|---|---|---|---|---|---|---|---|---|
| 'Pseudoalteromonas sp. CO348' (NZ SGPI00000000.1) | Pseudoalteromonas haloplanktis ATCC 14393 | 13.7 | [10.9 - 17.0] | 19.6 | [17.4 - 22.0] | 14 | [11.6 - 16.8] | 2.36 |
| 'Pseudoalteromonas sp. CO133X' (NZ SGPE00000000.1) | Pseudoalteromonas marina mano4 | 19.1 | [16.0 - 22.7] | 19.6 | [17.4 - 22.0] | 18.5 | [15.8 - 21.5] | 1.24 |
| 'Pseudoalteromonas sp. CO302Y' (NZ SGPF00000000.1) | Pseudoalteromonas marina mano4 | 19.1 | [16.0 - 22.7] | 19.6 | [17.4 - 22.0] | 18.5 | [15.8 - 21.5] | 1.23 |
| 'Pseudoalteromonas sp. CO342X' (NZ SGPH00000000.1) | Pseudoalteromonas arctica DSM 18437 | 13.5 | [10.7 - 16.8] | 19.6 | [17.4 - 22.0] | 13.8 | [11.4 - 16.6] | 4.21 |
| 'Pseudoalteromonas sp. CO342X' (NZ SGPH00000000.1) | Pseudoalteromonas rubra ATCC 29570 | 14.4 | [11.6 - 17.8] | 19.6 | [17.4 - 22.0] | 14.6 | [12.2 - 17.5] | 4.53 |
| 'Pseudoalteromonas sp. CO133X' (NZ SGPE00000000.1) | Pseudoalteromonas byunsanensis JCM 12483 | 14.4 | [11.5 - 17.8] | 19.6 | [17.4 - 22.0] | 14.5 | [12.1 - 17.4] | 1.59 |
| 'Pseudoalteromonas sp. CO109Y' (NZ SGPD00000000.1) | Pseudoalteromonas denitrificans DSM 6059 | 13.1 | [10.4 - 16.4] | 19.6 | [17.4 - 22.0] | 13.5 | [11.1 - 16.2] | 6.56 |
| 'Pseudoalteromonas sp. CO342X' (NZ SGPH00000000.1) | Pseudoalteromonas haloplanktis ATCC 14393 | 13.6 | [10.9 - 17.0] | 19.6 | [17.4 - 22.0] | 13.9 | [11.5 - 16.7] | 2.42 |
| 'Pseudoalteromonas sp. CO302Y' (NZ SGPF00000000.1) | Pseudoalteromonas peptidolytica NBRC 101021 | 14 | [11.2 - 17.3] | 19.5 | [17.3 - 21.9] | 14.2 | [11.8 - 17.0] | 1.55 |
| 'Pseudoalteromonas sp. CO342X' (NZ SGPH00000000.1) | Pseudoalteromonas marina mano4 | 13.7 | [10.9 - 17.0] | 19.5 | [17.3 - 21.9] | 13.9 | [11.5 - 16.8] | 3.61 |
| 'Pseudoalteromonas sp. CO109Y' (NZ SGPD00000000.1) | Pseudoalteromonas citrea NCIMB 1889 | 14.2 | [11.4 - 17.6] | 19.5 | [17.3 - 21.9] | 14.4 | [12.0 - 17.3] | 0.16 |
| 'Pseudoalteromonas sp. CO109Y' (NZ SGPD00000000.1) | 'Pseudoalteromonas sp. CO325X' (NZ SGPG00000000.1) | 13.9 | [11.1 - 17.2] | 19.5 | [17.3 - 21.9] | 14.1 | [11.7 - 16.9] | 5.97 |
| 'Pseudoalteromonas sp. CO133X' (NZ SGPE00000000.1) | Pseudoalteromonas peptidolytica NBRC 101021 | 14 | [11.2 - 17.3] | 19.5 | [17.3 - 21.9] | 14.2 | [11.8 - 17.0] | 1.55 |
| 'Pseudoalteromonas sp. CO342X' (NZ SGPH00000000.1) | Pseudoalteromonas prydzensis DSM 14232 | 13.9 | [11.1 - 17.2] | 19.5 | [17.3 - 21.9] | 14.1 | [11.7 - 16.9] | 2.11 |
| 'Pseudoalteromonas sp. CO348' (NZ SGPI00000000.1) | Pseudoalteromonas arctica DSM 18437 | 13.5 | [10.7 - 16.8] | 19.4 | [17.2 - 21.8] | 13.8 | [11.4 - 16.5] | 4.15 |
| 'Pseudoalteromonas sp. CO325X' (NZ SGPG00000000.1) | Pseudoalteromonas telluritireducens DSM 16098 | 13.7 | [10.9 - 17.0] | 19.4 | [17.2 - 21.8] | 13.9 | [11.5 - 16.8] | 6.33 |
| 'Pseudoalteromonas sp. CO348' (NZ SGPI00000000.1) | Pseudoalteromonas citrea NCIMB 1889 | 14.5 | [11.6 - 17.9] | 19.4 | [17.2 - 21.8] | 14.6 | [12.2 - 17.5] | 2.08 |
| 'Pseudoalteromonas sp. CO325X' (NZ SGPG00000000.1) | Pseudoalteromonas atlantica NBRC 103033 | 13.6 | [10.8 - 16.9] | 19.3 | [17.1 - 21.7] | 13.9 | [11.5 - 16.7] | 6.49 |
| 'Pseudoalteromonas sp. CO342X' (NZ SGPH00000000.1) | Pseudoalteromonas neustonica PAMC 28425 | 13.6 | [10.8 - 16.9] | 19.3 | [17.1 - 21.7] | 13.9 | [11.5 - 16.7] | 3.53 |
| 'Pseudoalteromonas sp. CO348' (NZ SGPI00000000.1) | Pseudoalteromonas neustonica PAMC 28425 | 13.6 | [10.8 - 16.9] | 19.2 | [17.0 - 21.6] | 13.9 | [11.5 - 16.7] | 3.48 |
| 'Pseudoalteromonas sp. CO348' (NZ SGPI00000000.1) | Pseudoalteromonas prydzensis DSM 14232 | 13.8 | [11.0 - 17.2] | 19.2 | [17.0 - 21.6] | 14.1 | [11.6 - 16.9] | 2.05 |
| 'Pseudoalteromonas sp. CO325X' (NZ SGPG00000000.1) | Pseudoalteromonas prydzensis DSM 14232 | 13.7 | [10.9 - 17.0] | 19.2 | [17.1 - 21.6] | 13.9 | [11.5 - 16.7] | 6.1 |
| 'Pseudoalteromonas sp. CO325X' (NZ SGPG00000000.1) | Pseudoalteromonas arctica DSM 18437 | 13.5 | [10.7 - 16.8] | 19.2 | [17.0 - 21.6] | 13.8 | [11.4 - 16.6] | 8.2 |
| 'Pseudoalteromonas sp. CO342X' (NZ SGPH00000000.1) | Pseudoalteromonas denitrificans DSM 6059 | 12.9 | [10.2 - 16.2] | 19.1 | [16.9 - 21.5] | 13.3 | [10.9 - 16.0] | 8.53 |
| 'Pseudoalteromonas sp. CO342X' (NZ SGPH00000000.1) | Vibrio panuliri LBS2 | 12.8 | [10.1 - 16.1] | 19.1 | [16.9 - 21.5] | 13.2 | [10.8 - 15.9] | 1.85 |

| | | | | | | | | |
|---|---|---|---|---|---|---|---|---|
| 'Pseudoalteromonas sp. CO325X' (NZ SGPG00000000.1) | Pseudoalteromonas lipolytica CGMCC 1.8499 | 13.8 | [11.0 - 17.2] | 19.1 | [17.0 - 21.5] | 14.1 | [11.7 - 16.9] | 5.77 |
| 'Pseudoalteromonas sp. CO325X' (NZ SGPG00000000.1) | Pseudoalteromonas denitrificans DSM 6059 | 12.8 | [10.1 - 16.1] | 19 | [16.8 - 21.4] | 13.2 | [10.9 - 16.0] | 12.53 |
| 'Pseudoalteromonas sp. CO325X' (NZ SGPG00000000.1) | Pseudoalteromonas marina mano4 | 13.6 | [10.8 - 16.9] | 19 | [16.8 - 21.4] | 13.9 | [11.5 - 16.7] | 7.6 |

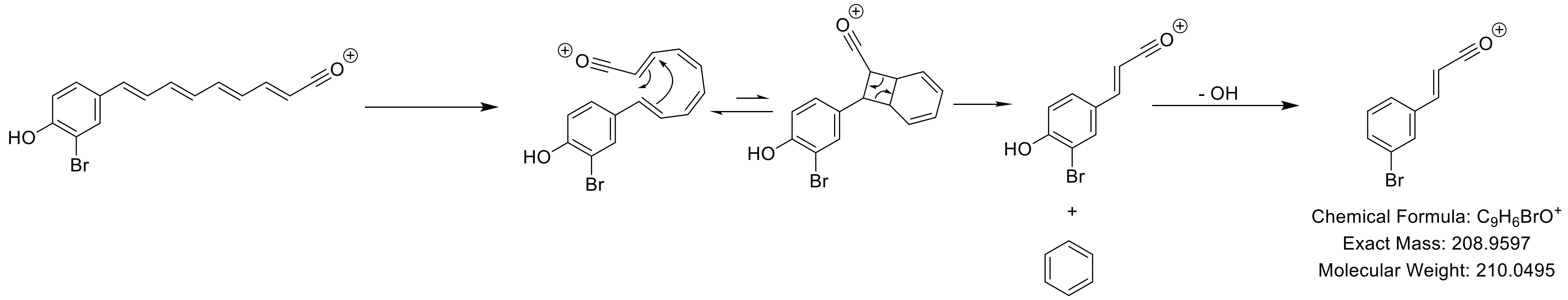


**Figure S1. Aromatic elimination of aryl polyenes**

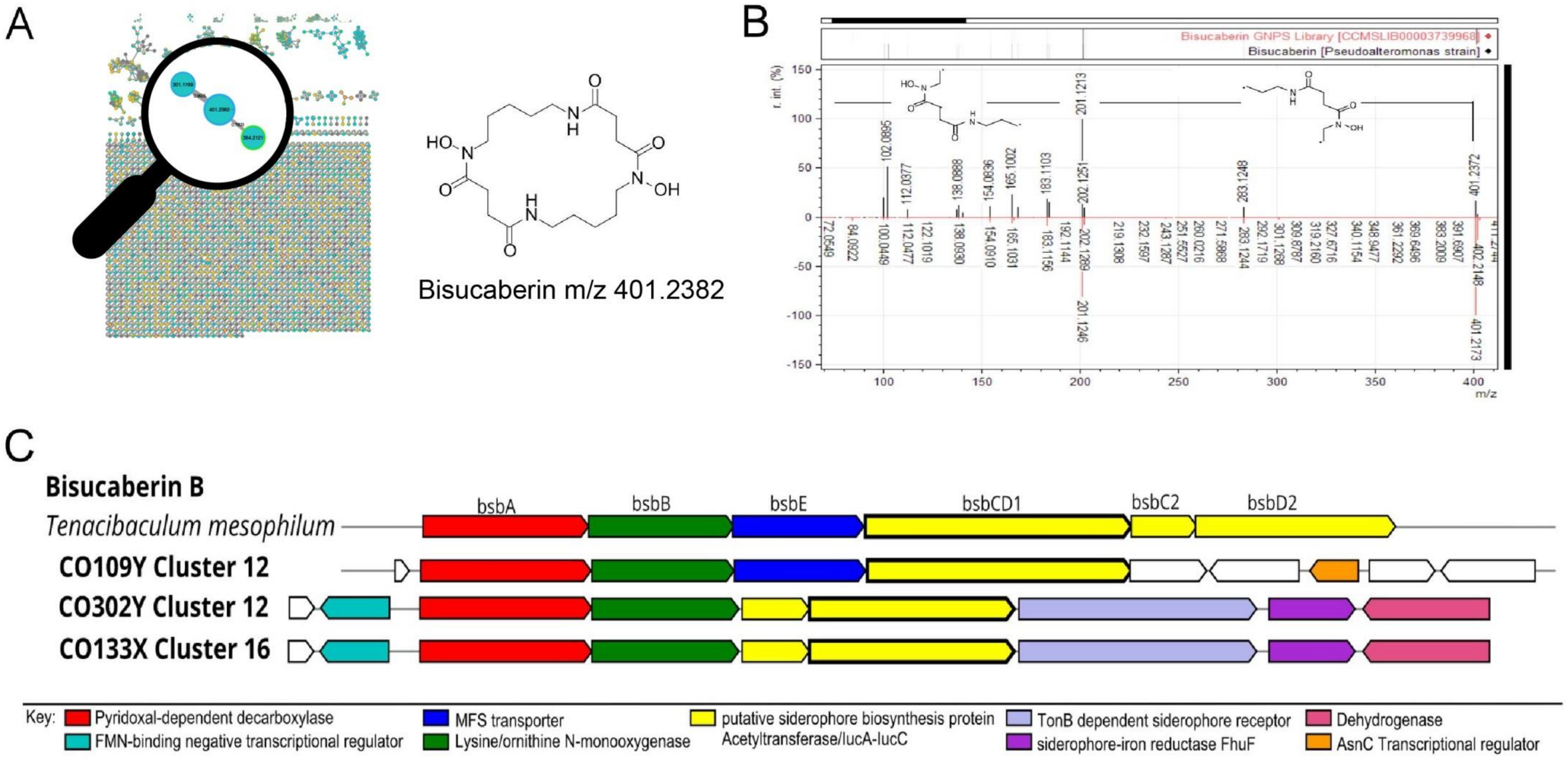


**Figure S2. Bisucaberin** A) Node of bisucaberin in the molecular network and its molecular structure. B) MS/MS fragmentation patterns of Bisucaberin. C) Synteny map of bisucaberin B BGC (*Tenacibaculum mesophilum* NBCR 16307) compared with clusters of non-pigmented strains, all homologous genomic regions were identified by MultiGeneBlast. The most similar BGC is showed immediately below the cluster. The core biosynthetic genes are shown in bold.

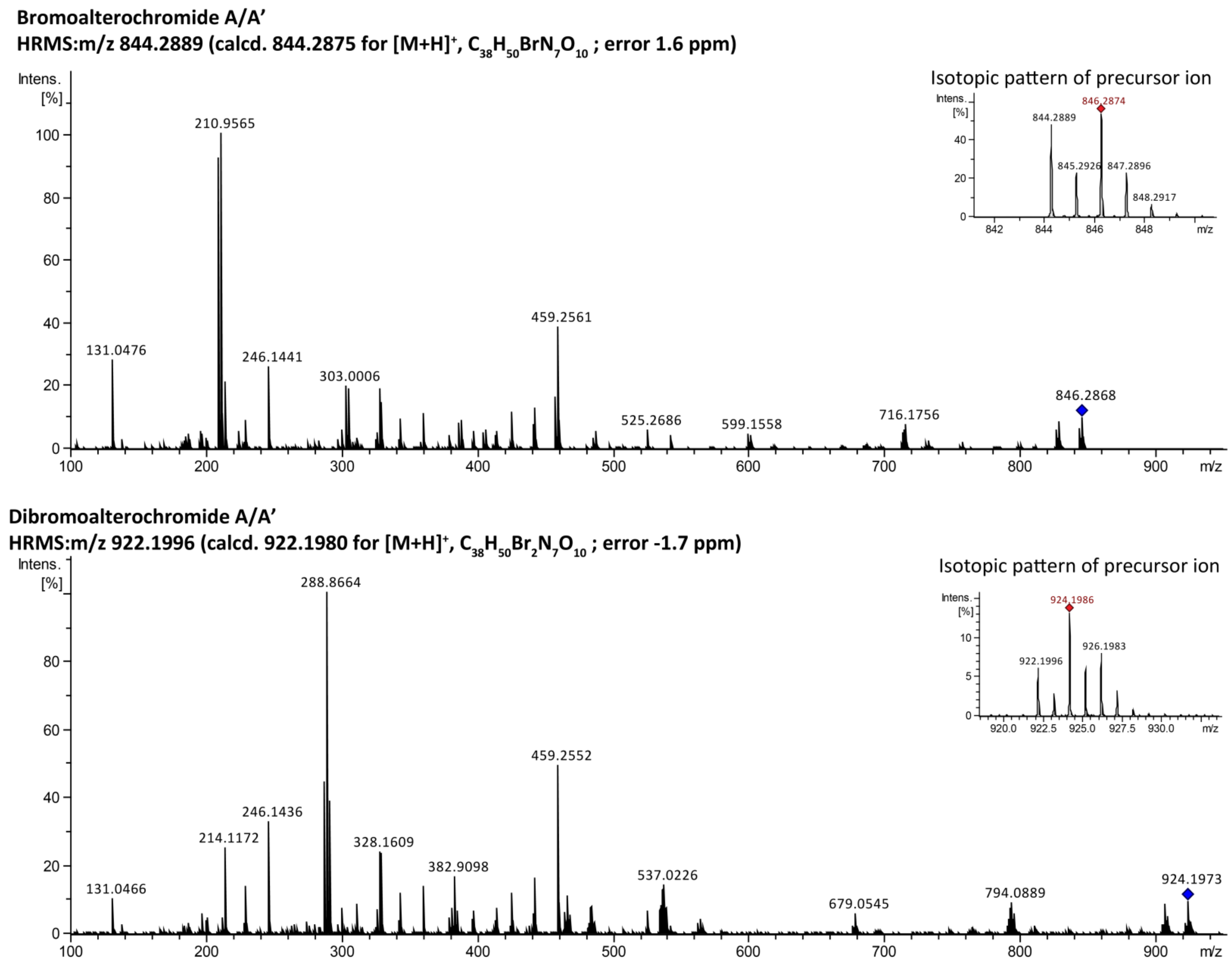


**Figure S3.** MS/MS spectra for bromoalterochromide A/A′ (**1**,**2**) and dibromoalterochromide A/A′ (**3**,**4**).

**Bromoalterochromide B/B′**

**HRMS:m/z 870.3061 (calcd. 870.3032 for $[M+H]^+$, $C_{40}H_{53}BrN_7O_{10}$ ; error 3.4 ppm)**

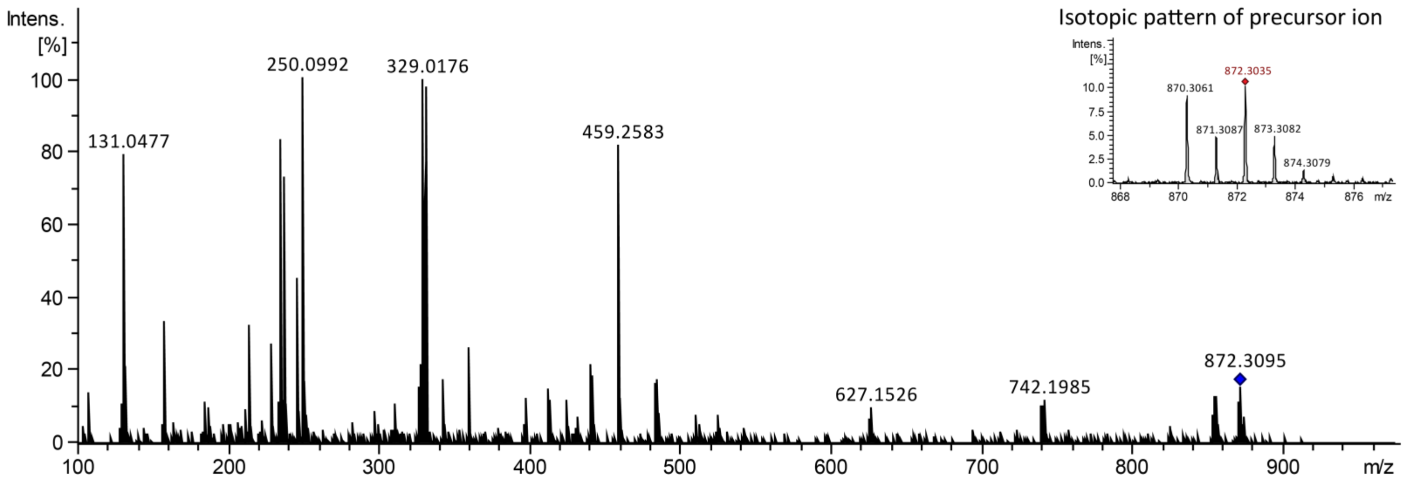


**Dibromoalterochromide B/B′**

**HRMS:m/z 948.2175 (calcd. 948.2137 for $[M+H]^+$, $C_{40}H_{52}Br_2N_7O_{10}$ ; error -4.1 ppm)**

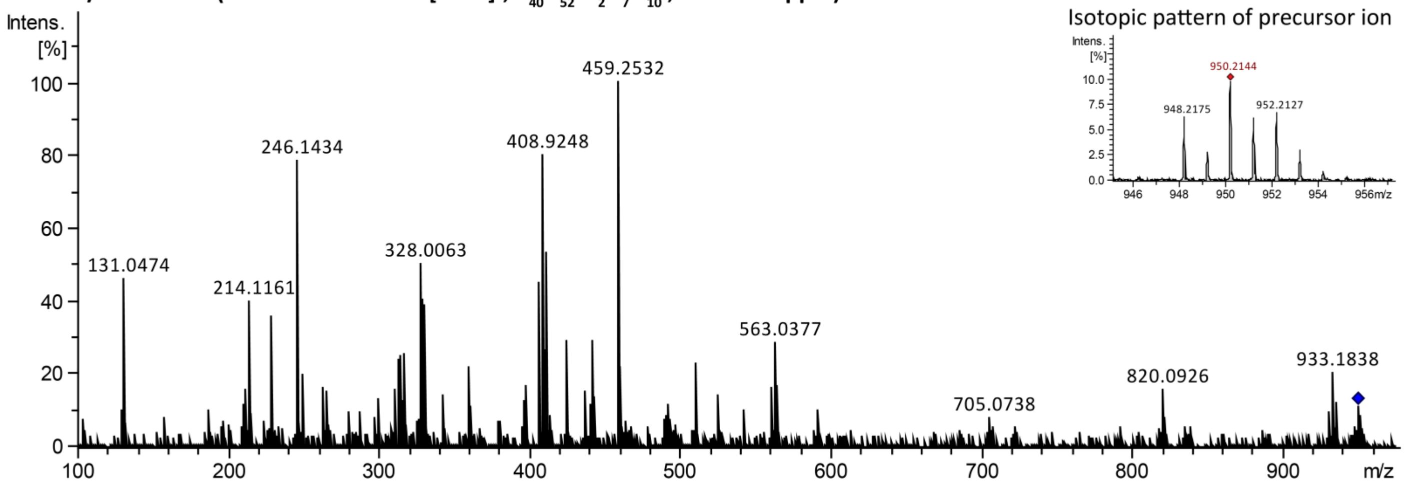


**Figure S4.** MS/MS spectra for bromoalterochromide B/B′ (**5**,**6**) and dibromoalterochromide B/B′ (**7**,**8**).

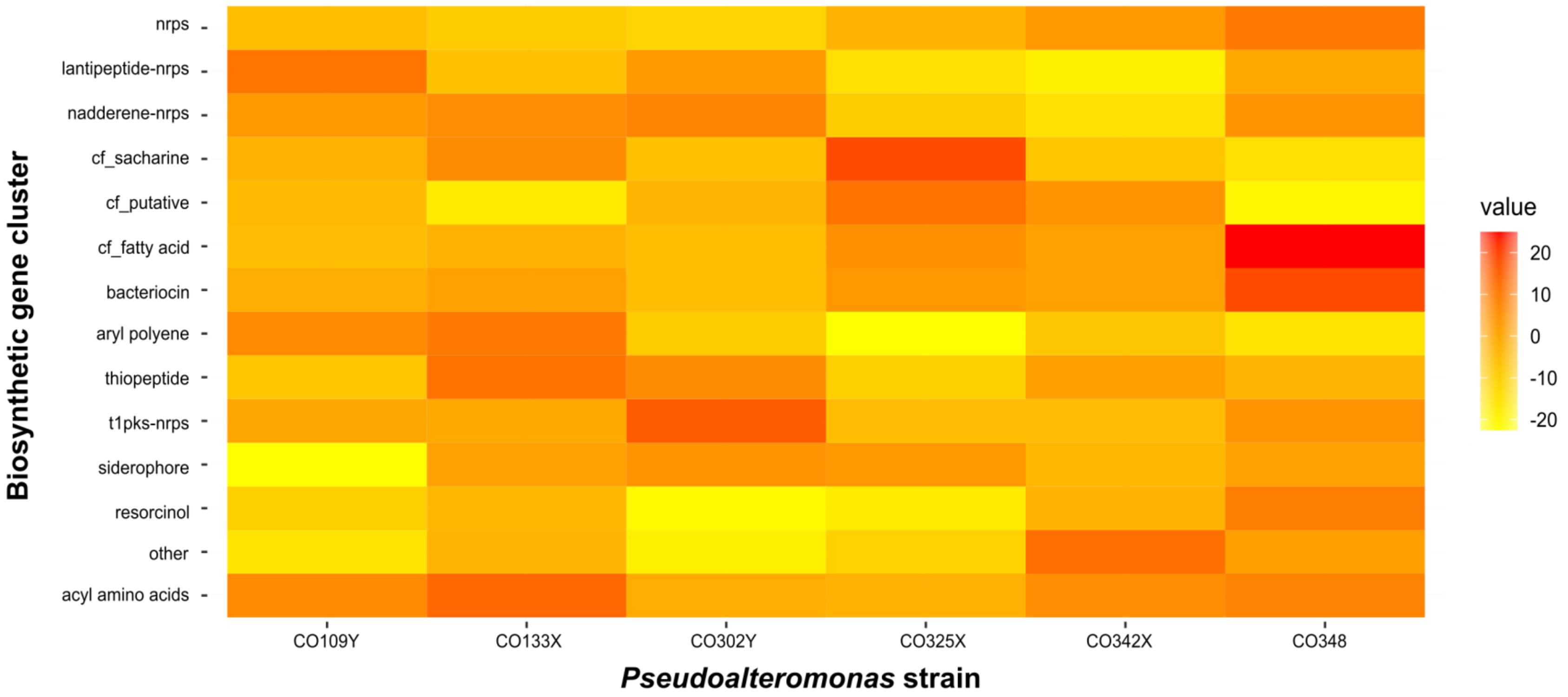


**Figure S5.** Heatmap containing BGCs categories predicted by AntiSMASH in *Pseudoalteromonas* strains genomes.

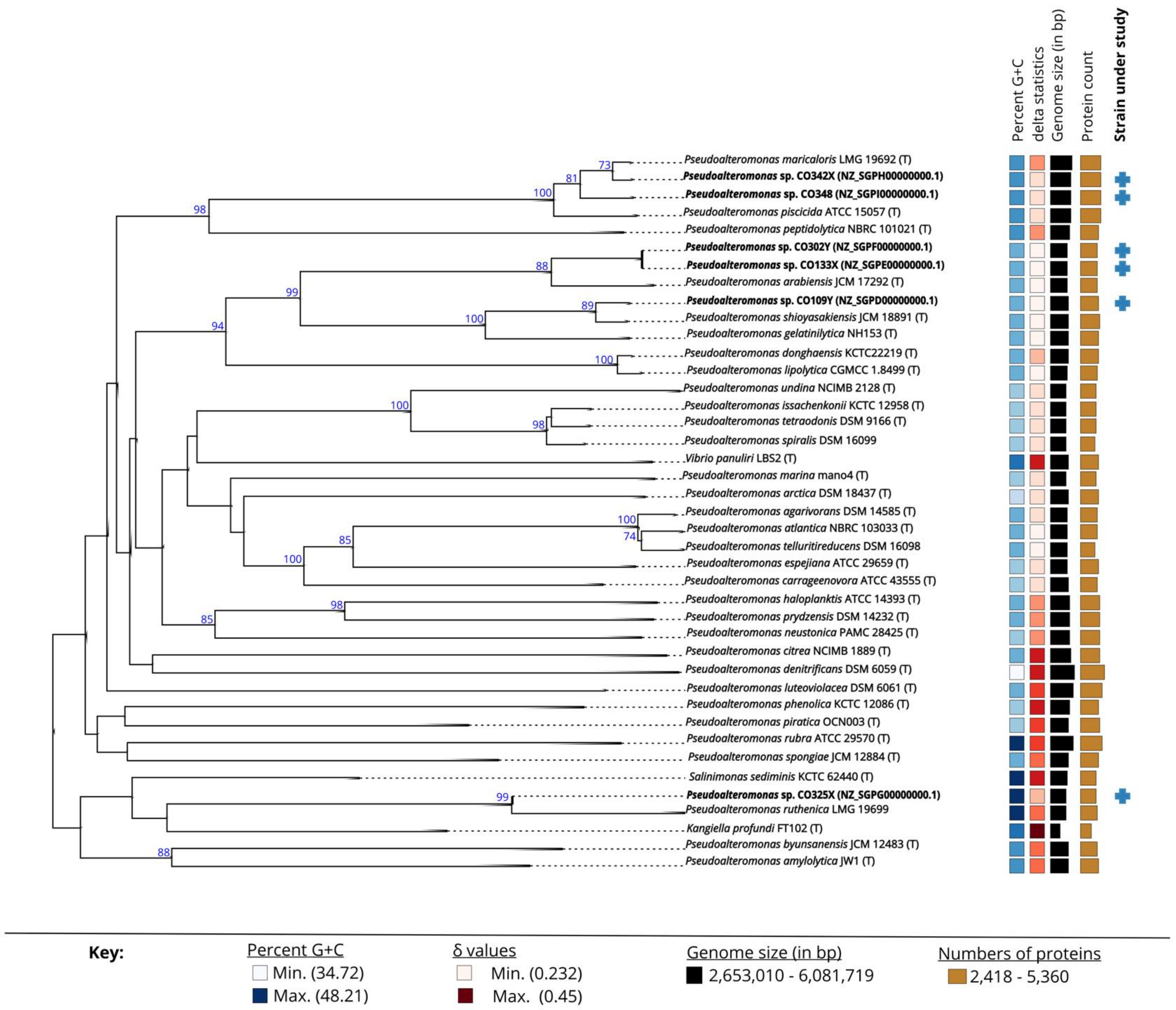


**Figure S6.** Genome Blast Distance Phylogeny (GBDP) of *Pseudoalteromonas* strains from Coiba. Tree inferred with FastME 2.1.6.1 from GBDP distances calculated from genome sequences. The branch lengths are scaled in terms of GBDP distance formula d5. The numbers above branches are GBDP pseudo-bootstrap support values (> 70% are shown next to its respective branch). Leaf labels are annotated by affiliation to species, genomic G+C content, δ values, overall genome sequence length, and number of proteins. *Pseudoalteromonas* strains genomes from Coiba National Park are highlighted in bold and with a blue cross in the last annotations column.
